# A list of land plants of Parque Nacional do Caparaó, Brazil, highlights the presence of sampling gaps within this protected area

**DOI:** 10.3897/BDJ.8.e59664

**Published:** 2020-12-31

**Authors:** Marina M Moreira, Tatiana T Carrijo, Anderson G Alves-Araújo, Alessandro Rapini, Alexandre Salino, Aline D Firmino, Aline P Chagas, Ana F A Versiane, André M A Amorim, Andrews V S da Silva, Amélia C Tuler, Ariane L Peixoto, Bethina S Soares, Braz A P Cosenza, Camila N Delgado, Claudia R Lopes, Christian Silva, Daniel E F Barbosa, Daniele Monteiro, Danilo Marques, Dayvid R Couto, Diego R Gonzaga, Eduardo Dalcin, Elton John de Lirio, Fabrício S Meyer, Fátima R G Salimena, Felipe A. Oliveira, Filipe S Souza, Fernando B Matos, Gabriel Depiantti, Guilherme M Antar, Gustavo Heiden, Henrique M Dias, Hian C F Sousa, Isabel T F V Lopes, Isis M Rollim, Jaquelini Luber, Jefferson Prado, Jimi N Nakajima, João Lanna, João Paulo F Zorzanelli, Joelcio Freitas, José F A Baumgratz, Jovani B S Pereira, Juliana R P M Oliveira, Kelly Antunes, Lana S Sylvestre, Leandro C Pederneiras, Leandro Freitas, Leandro L Giacomin, Leonardo D Meireles, Leonardo N Silva, Luciana C Pereira, Luís Alexandre E Silva, Luiz Menini Neto, Marcelo Monge, Marcelo L O Trovó, Marcelo Reginato, Marcos E G Sobral, Mario Gomes, Mário L Garbin, Marli P Morim, Nayara D Soares, Paulo H E Labiak, Pedro L Viana, Pedro H Cardoso, Pedro L R Moraes, Pedro B Schwartsburd, Quélita S Moraes, Raquel F Zorzanelli, Renara Nichio-Amaral, Renato Goldenberg, Samyra G Furtado, Thamara Feletti, Valquíria F Dutra, Vinícius R Bueno, Vinícius A O Dittrich, Rafaela C Forzza

**Affiliations:** 1 Universidade Federal do Espírito Santo, Vitória, Brazil Universidade Federal do Espírito Santo Vitória Brazil; 2 Jardim Botânico do Rio de Janeiro, Rio de Janeiro, Brazil Jardim Botânico do Rio de Janeiro Rio de Janeiro Brazil; 3 Universidade Estadual de Feira de Santana, Feira de Santana, Bahia, Brazil Universidade Estadual de Feira de Santana Feira de Santana, Bahia Brazil; 4 Universidade Federal de Minas Gerais, Belo Horizonte, Brazil Universidade Federal de Minas Gerais Belo Horizonte Brazil; 5 Fundação Espírito-Santense de Tecnologia, Vitória, Brazil Fundação Espírito-Santense de Tecnologia Vitória Brazil; 6 Secretaria de Desenvolvimento da Cidade e Meio Ambiente, Cariacica, Brazil Secretaria de Desenvolvimento da Cidade e Meio Ambiente Cariacica Brazil; 7 Universidade Estadual de Campinas, Campinas, Brazil Universidade Estadual de Campinas Campinas Brazil; 8 Universidade Estadual de Santa Cruz, Ilhéus, Brazil Universidade Estadual de Santa Cruz Ilhéus Brazil; 9 Universidade Federal do Rio de Janeiro, Rio de Janeiro, Brazil Universidade Federal do Rio de Janeiro Rio de Janeiro Brazil; 10 Instituto Nacional da Mata Atlântica, Santa Teresa, Brazil Instituto Nacional da Mata Atlântica Santa Teresa Brazil; 11 Universidade do Estado de Minas Gerais, Belo Horizonte, Brazil Universidade do Estado de Minas Gerais Belo Horizonte Brazil; 12 Universidade Federal de Juiz de Fora, Juiz de Fora, Brazil Universidade Federal de Juiz de Fora Juiz de Fora Brazil; 13 Universidade Federal de Alfenas, Alfenas, Brazil Universidade Federal de Alfenas Alfenas Brazil; 14 Universidade do Estado de Santa Catarina, Laguna, Brazil Universidade do Estado de Santa Catarina Laguna Brazil; 15 Universidad Nacional del Nordeste - Consejo Nacional de Investigaciones Científicas y Técnicas, Corrientes, Argentina Universidad Nacional del Nordeste - Consejo Nacional de Investigaciones Científicas y Técnicas Corrientes Argentina; 16 Universidade Estadual do Norte Fluminense Darcy Ribeiro, Campos dos Goytacazes, Brazil Universidade Estadual do Norte Fluminense Darcy Ribeiro Campos dos Goytacazes Brazil; 17 Universidade de São Paulo, São Paulo, Brazil Universidade de São Paulo São Paulo Brazil; 18 STCP Engenharia de Projetos, Curitiba, Brazil STCP Engenharia de Projetos Curitiba Brazil; 19 Universidade Federal do Paraná, Curitiba, Brazil Universidade Federal do Paraná Curitiba Brazil; 20 Embrapa Clima Temperado, Pelotas, Brazil Embrapa Clima Temperado Pelotas Brazil; 21 Universidade Federal de Uberlândia, Uberlândia, Brazil Universidade Federal de Uberlândia Uberlândia Brazil; 22 Universidade Estadual Paulista Júlio de Mesquita Filho, São José do Rio Preto, Brazil Universidade Estadual Paulista Júlio de Mesquita Filho São José do Rio Preto Brazil; 23 Universidade Estadual de Feira de Santana, Feira de Santana, Brazil Universidade Estadual de Feira de Santana Feira de Santana Brazil; 24 Instituto de Botânica, São Paulo, Brazil Instituto de Botânica São Paulo Brazil; 25 Universidade Federal do Oeste do Pará, Santarém, Brazil Universidade Federal do Oeste do Pará Santarém Brazil; 26 Universidade Federal do Rio Grande do Sul, Porto Alegre, Brazil Universidade Federal do Rio Grande do Sul Porto Alegre Brazil; 27 Universidade Federal de São João del-Rei, São João del-Rei, Brazil Universidade Federal de São João del-Rei São João del-Rei Brazil; 28 Universidade Federal do Parana, Curitiba, Brazil Universidade Federal do Parana Curitiba Brazil; 29 Museu Paraense Emílio Goeldi, Belém, Brazil Museu Paraense Emílio Goeldi Belém Brazil; 30 Universidade Estadual Paulista Júlio de Mesquita Filho, Rio Claro, Brazil Universidade Estadual Paulista Júlio de Mesquita Filho Rio Claro Brazil; 31 Universidade Federal de Viçosa, Viçosa, Brazil Universidade Federal de Viçosa Viçosa Brazil

**Keywords:** Atlantic Forest, conservation, endemism, plant richness, threatened species.

## Abstract

**Background:**

Brazilian protected areas are essential for plant conservation in the Atlantic Forest domain, one of the 36 global biodiversity hotspots. A major challenge for improving conservation actions is to know the plant richness, protected by these areas. Online databases offer an accessible way to build plant species lists and to provide relevant information about biodiversity. A list of land plants of “Parque Nacional do Caparaó” (PNC) was previously built using online databases and published on the website "Catálogo de Plantas das Unidades de Conservação do Brasil." Here, we provide and discuss additional information about plant species richness, endemism and conservation in the PNC that could not be included in the List. We documented 1,791 species of land plants as occurring in PNC, of which 63 are cited as threatened (CR, EN or VU) by the Brazilian National Red List, seven as data deficient (DD) and five as priorities for conservation. Fifity-one species were possible new ocurrences for ES and MG states.

**New information:**

"Parque Nacional do Caparaó" houses 8% of the land plant species endemic to the Brazilian Atlantic Forest, including 6% of its angiosperms, 31% of its lycophytes and ferns and 14% of its avascular plants. Twelve percent of the threatened species listed for the State of Espírito Santo and 7% listed for the State of Minas Gerais are also protected by PNC. Surprisingly, 79% of the collections analysed here were carried out in Minas Gerais, which represents just 21% of the total extension of the Park. The compiled data uncover a huge botanical collection gap in this federally-protected area.

## Introduction

The Atlantic Forest is recognised worldwide for its high biological diversity, high rates of endemism and great threat (with more than 70% of its original area devastated) and which is considered one of 36 global biodiversity hotspots (Marques and Grelle 2020). It has been estimated that this phytogeographic domain contains 17,776 species of land plants with an endemism rate of 56% (Flora do Brasil under construction 2020). Only 28% of the Atlantic Forest original area remains and its area consists mainly of edge-affected or secondary vegetation disconnected from larger remnants due to the long and intense disturbances it has experienced throughout its history (Rezende et al. 2018). There are 1,437 protected areas (PAs) in the Atlantic Forest, including strictly-protected areas (477) and areas managed for sustainable use (960) (MMA 2020). However, only 30% of the total vegetation cover is located within PAs, of which 9% are strictly protected (Rezende et al. 2018) and little is known about the plant species that are protected within them (Oliveira et al. 2017).

In the past 20 years, virtual tools have become more popular by sharing information from herbaria all over the world to contribute and to facilitate voucher identification, such as the Global Biodiversity Information Facility (GBIF; http://www.gbif.org), REFLORA (http://reflora.jbrj.gov.br) and INCT-Splink (http://inct.splink.org.br). The availability of biodiversity data on the internet has not only increased communication amongst herbaria, it has also provided information for documenting biodiversity and its distribution in space and time and, thus, has served as a backbone for developing environmental policies (Maldonado et al. 2015, Nualart et al. 2017, Oliveira et al. 2017). In addition, as the Atlantic Forest is one of the best-sampled phytogeographic domains in Brazil, the use of online databases can assist in documenting biodiversity and revealing temporal, spatial and taxonomic gaps in knowledge (Oliveira et al. 2017, Oliveira et al. 2019, Colli‐Silva et al. 2020).

“Catálogo de Plantas das Unidades de Conservação do Brasil” (https://catalogo-ucs-brasil.jbrj.gov.br/) is a digital platform created in 2018 with the aim to host lists of plant species that occur within Brazilian protected areas. Currently, the catalogue contains plant lists for five PAs from different Brazilian phytogeographic domains, such as the Amazon, Caatinga and Atlantic Forest. The plant list for “Parque Nacional do Itatiaia” (PNI) was the first made available on this platform (Carrijo et al. 2018), while the list for “Parque Nacional do Caparaó” (PNC) was the most recent (Carrijo et al. 2020). In addition to access to lists of species and vouchers, the website also provides the threat category of species, information on the occurrence of non-native and native species and a list of priority species for conservation for each protected area.

“Parque Nacional do Caparaó” (PNC), created in 1961 and effectively implemented in 1979, aims to protect areas of the Atlantic Forest domain (Santos 2004, ICMBio 2015). The first scientific expeditions to the area now occupied by the PNC were carried out by Wilhelm Schwacke in 1880 and by the Belgian-Brazilian Mission in 1922 (Massart et al. 1929). The first floristic inventory, however, was carried out by Alexander Curt Brade in 1942 and this recorded 259 species of plants of 59 families (Brade 1942). Other Botanists who contributed significantly to the knowledge of the local flora were Alexandre Salino, Braz AP Cosenza, Leopoldo Krieger, Lúcio de Souza Leoni and Vinicius Castro Souza (Santos 2004). Despite this intense collection effort, knowledge of the flora of PNC is dispersed in numerous publications on specific groups or families of plants (Aguiar and Marques 2001, Souza and Souza 2002, Romão and Souza 2003, Leoni and Chautems 2004, Mazine and Souza 2008, Leoni 2009a, Leoni 2009b, Forster and Souza 2013, Góes-Neto et al. 2015, Machado et al. 2016, Araújo et al. 2018, Góes-Neto and Salino 2018, Cardoso et al. 2019, Camelo et al. 2020). There remains, however, a lack of a single source providing access to reliable information about plant species protected by this PA. Here, we provide and discuss additional information about species richness, endemism and conservation in PNC that could not be provided in the plant list for PNC previously published in the catalogue (after Carrijo et al. 2020).

## Sampling methods

### Study extent

**Figure 1.**
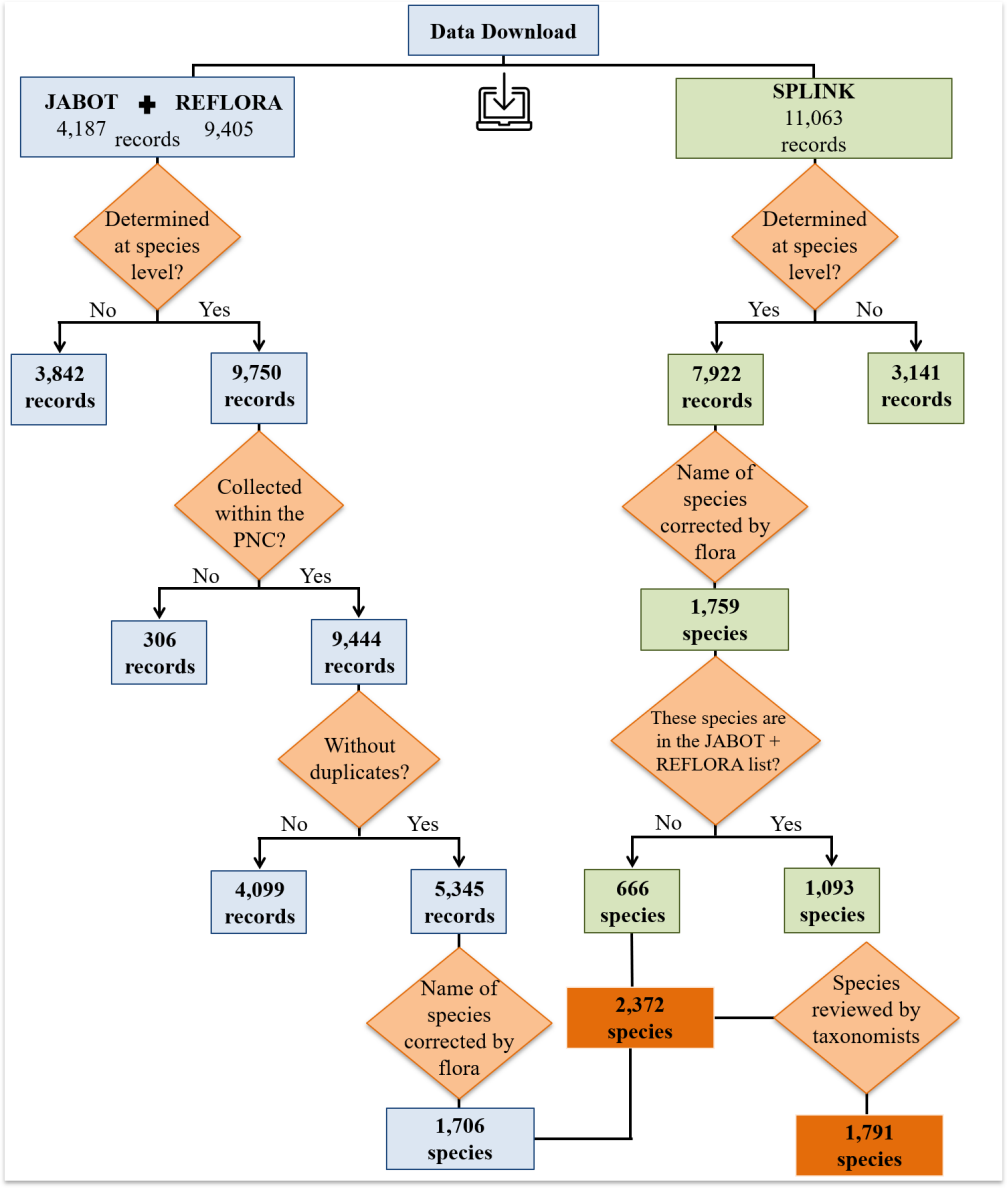
Stages of data cleaning performed in R software to obtain a list of land plants of “Parque Nacional do Caparaó,” Brazil, from the online databases.

A list of all plant specimens from PNC in three databases was compiled from downloads: JABOT (“Jardim Botânico do Rio de Janeiro”, JBRJ, http://www.jbrj.gov.br/jabot), REFLORA (“Herbário Virtual Reflora”, http://reflora.jbrj.gov.br) and Splink (“INCT Herbário Virtual da Flora e dos Fungos”, http://inct.splink.org.br). Searches were performed in each database on 15 April 2019, using the following filters: (1) locality = Caparaó and (2) locality = Caparao (without special characters). These searches led to a total of 24,655 specimens (JABOT = 4,187; REFLORA = 9,405; Splink = 11,063; Fig. 1).

To obtain a list of species with currently-accepted nomenclature, we manually selected all specimens identified to the species level, leading to the following: JABOT determined = 3,113, undetermined = 1,074; REFLORA determined = 6,637, undetermined = 2,768; and Splink determined = 7,922, undetermined = 3,141 (Fig. 1). We also removed specimens that had a locality that did not belong to the area covered by the PNC, as well as duplicates (based on the catalogue code, collector name and number and the year in which the sample was collected) (Fig. 1). We then corrected and updated species names and determined their threat categories using the function *get.taxa* from the *flora* package (Carvalho 2017) of R software v. 3.5.3 (R Development Core Team 2019). We used this function to compare the names in our list with those in Flora do Brasil under construction (2020) and determined the threat category for each species according to the Red List Authority for plants in Brazil - CNCFLora (http://www.cncflora.jbrj.gov.br/portal) (Fig. 1). Introduced species were not recovered by the function *get.taxa* and so these specimens had to be reviewed manually. After these corrections, taxonomists checked the preliminary list of 2,372 species virtually, using images available in the online databases (Fig. 1). When a taxonomist modified a plant species name, at least one specimen of that species was updated in the Herbarium of “Jardim Botânico do Rio de Janeiro” (RB, acronym from Thiers 2020) and its database JABOT, as well as in the REFLORA database, but not in Splink. Infraspecific taxonomic categories were not considered, nor were hybrids.

To assess the most collected sites in the Park, we built a word cloud, based on the name of locations where specimens were collected. We did not include broad locations in the word cloud, such as “Serra do Caparaó” and “Parque Nacional do Caparaó”, since they are not informative. We constructed the word cloud using the function *wordcloud* of the *wordcloud* package version 2.6 (Fellows 2018).

To evaluate whether species were native or non-native and endemic or non-endemic to Brazil and to assign a threat category, we used information from Flora do Brasil 2020 (under construction; http://floradobrasil.jbrj.gov.br) and the Red List Authority for plants in Brazil - CNCFLora (http://www.cncflora.jbrj.gov.br/portal). In the case of species not included in the Flora do Brasil 2020 database, threat category and origin (native/non-native) were obtained from taxonomists. We considered as non-native all species indicated as cultivated or naturalised by the Flora do Brasil 2020 database. We also assessed whether species of PNC were on the threatened species lists in the States of Espírito Santo (Fraga et al. 2019) and Minas Gerais (COPAM-MG 2008). We classified species as endemic to the Atlantic Forest when their distribution is restricted to the Atlantic Forest phytogeographic domain. We obtained this information from the Flora do Brasil 2020 database through the function *get_domains* from the *flora* package (Carvalho 2017). This classification also included species non-endemic to Brazil as the Atlantic Forest occurs in the countries of Argentina and Paraguay. We classified species as rare in Brazil, based on the publication of Giulietti et al. (2009).

We classified species as a priority for conservation when they had a single record collected before 1970 (Briggs and Leigh 1988) and was simultaneously categorised as Critically Endangered (CR), Vulnerable (VU), Endangered (EN) or Data Deficient (DD), according to Red List Authority for plants in Brazil - CNCFLora (http://www.cncflora.jbrj.gov.br/portal).

## Geographic coverage

### Description

The PNC encompasses a total area of 31,853.12 ha (20° 37’ to 20° 19’ S, 41° 43’ to 41° 55’ W), of which 79% is in Espírito Santo (ES) and 21% in Minas Gerais (MG), both in southeast Brazil (ICMBio 2015). Being the second largest conservation unit within ES (Fraga et al. 2019), it encompasses five of its Municipalities (Divino de São Lourenço, Dores do Rio Preto, Ibitirama, Irupi and Iúna), plus four in MG (Alto Caparaó, Alto Jequitibá, Caparaó and Espera Feliz) (ICMBio 2015). Located in “Serra da Mantiqueira”, PNC has a wide altitudinal gradient (630 to 2,892 m a.s.l.), with “Pico da Bandeira” (2,892 m a.s.l.) being its highest point and the third highest peak in Brazil (Fig. 2a) (ICMBio 2015).

Mean annual rainfall in PNC is 1,481.1 mm, with the greatest rainfall in January (mean 316.7 mm) and the lowest in July (mean 16.2 mm; data are mean rainfall for 1974–2003; ICMBio 2015). The mean annual temperature is 11°C, with a minimum of 2.5°C in July and a maximum of 31°C in December (data are mean temperatures between December 2004 and December 2005 at 2,400 m a.s.l.; Rodrigues et al. 2009). The vegetation of PNC comprises different phytophysiognomies, including dense ombrophilous montane forest, dense ombrophilous high-montane forest, seasonal semi-deciduous montane forest and high-altitude grassland (*campos de altitude*) (Fig. 2b, c, d; ICMBio 2015).

### Coordinates

-20° 37’ and -20° 19’ S Latitude; -41° 55’ and -41° 43’ Longitude.

## Taxonomic coverage

### Description

The plant list for “Parque Nacional do Caparaó” contains a total of 1,791 species (Suppl. material 1) of 715 genera and 198 botanical families, with 1,292 angiosperms (500 genera/119 families), 38 lycophytes (9/3), 263 ferns (89/20) and 198 avascular plants (i.e. Antocerotophyta, Bryophyta and Marchantiophyta; 117/56) (Figs 3, 4). We found no records for gymnosperms in PNC in the online databases; however, a recent and unpublished study (Araújo 2016) recorded *Podocarpus lambertii* Klotzsch ex Endl. in PNC. During expeditions to the Park, we only observed some individuals of *Araucaria angustifolia* (Bertol.) Kuntze (Fig. 3f), but this species is usually planted in montane areas of south-eastern Brazil and there are doubts about its natural occurrence in several localities, with it being considered regionally extinct in ES (Tuler et al. 2019).

The richest families in PNC are: Orchidaceae with 152 species, Asteraceae (139), Poaceae (87), Melastomataceae (74), Solanaceae (53), Dryopteridaceae (52), Fabaceae (51), Myrtaceae (50), Polypodiaceae (47) and Bromeliaceae and Rubiaceae (both with 44). These families comprise 44% (i.e. 793 species) of the species found in PNC (Fig. 5a, b, c). Forty-nine families are represented by a single species. The richest genera are: *Solanum* with 33 species, *Elaphoglossum* (29), *Leandra* (26), *Baccharis* and *Piper* (both with 24), *Myrcia* (23), *Asplenium* (22), *Miconia* (21), *Amauropelta* and *Habenaria* (both with 19), *Peperomia* and *Phlegmariurus* (both with 17) and *Vriesea* (16). These genera comprise 16% (i.e. 290 species) of the species found in PNC (Fig. 5d, e, f). A total of 388 genera are represented by a single species.

**Figure 6.**
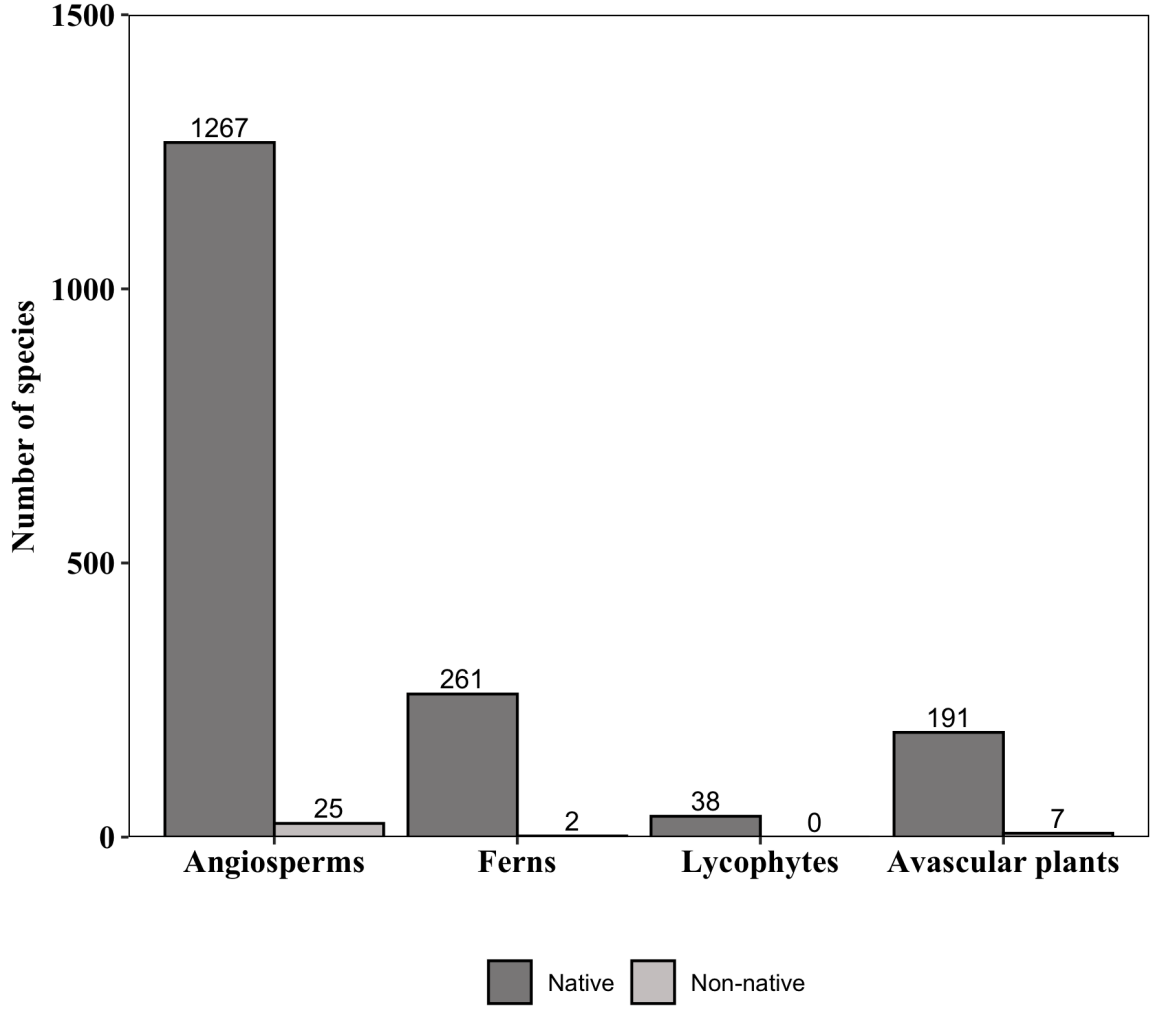
Native and non-native species to Brazil of major groups of plants that occur in “Parque Nacional do Caparaó,” Brazil. Values above the bars indicate the number of species.

The list for PNC includes 1,757 native and 34 non-native species to Brazil (Fig. 6). Most of the native species are angiosperms (72%), followed by ferns (15%), avascular plants (11%) and lycophytes (2%; Fig. 6). All lycophyte species occurring in PNC are native. Amongst non-native species, angiosperms accounted for 73%, followed by avascular plants with 21% and ferns with 6% (Suppl. material 1). Asteraceae (8 species) and Poaceae (5) had the highest number of non-native species in PNC (Suppl. material 1). Thirteen families are represented by a single non-native species (Suppl. material 1).

**Figure 7.**
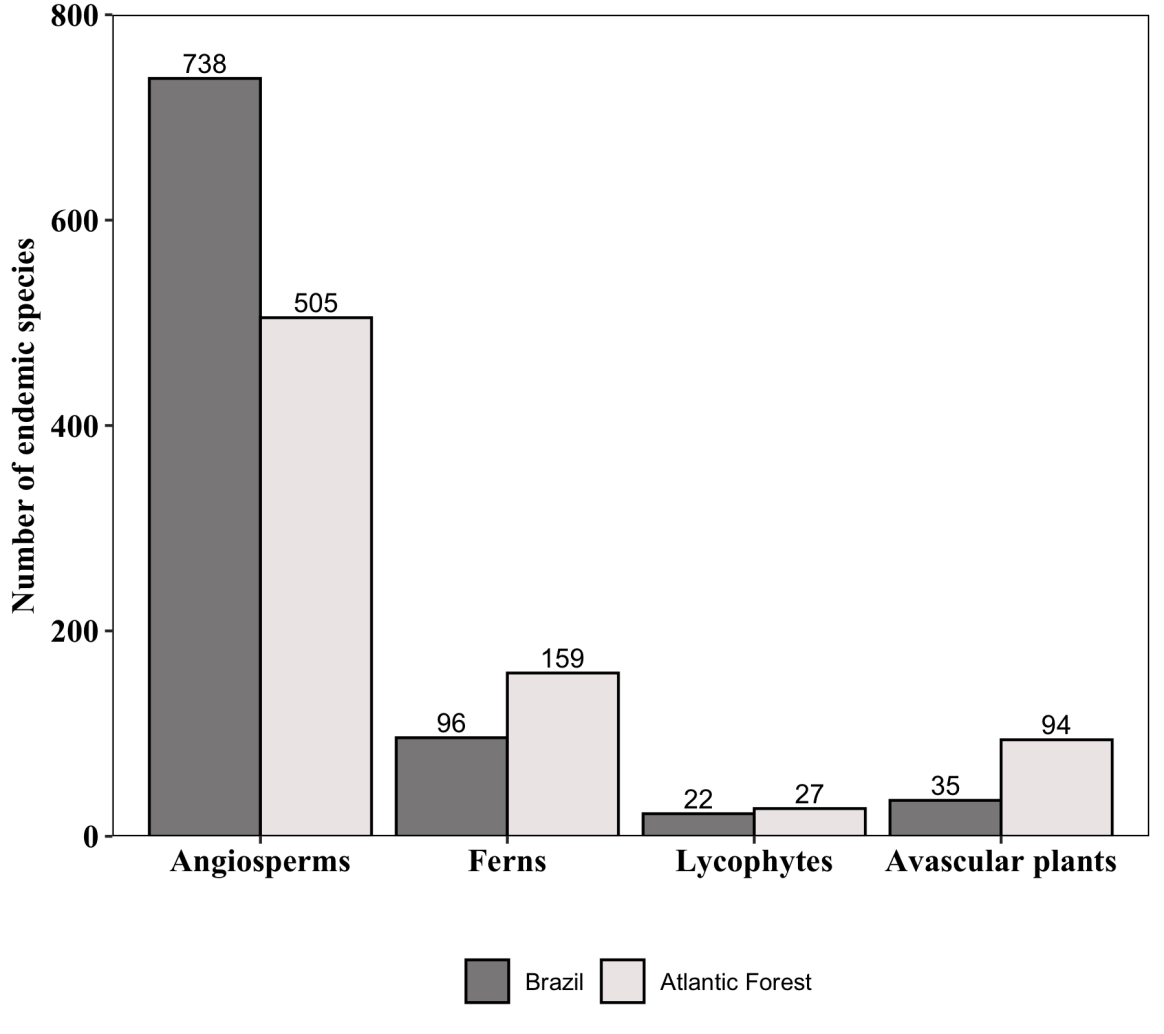
Number of endemic species to Brazil and to the Atlantic Forest of major groups of plants that occur in the “Parque Nacional do Caparaó,” Brazil. Values above the bars indicate the number of species.

**Figure 8.**
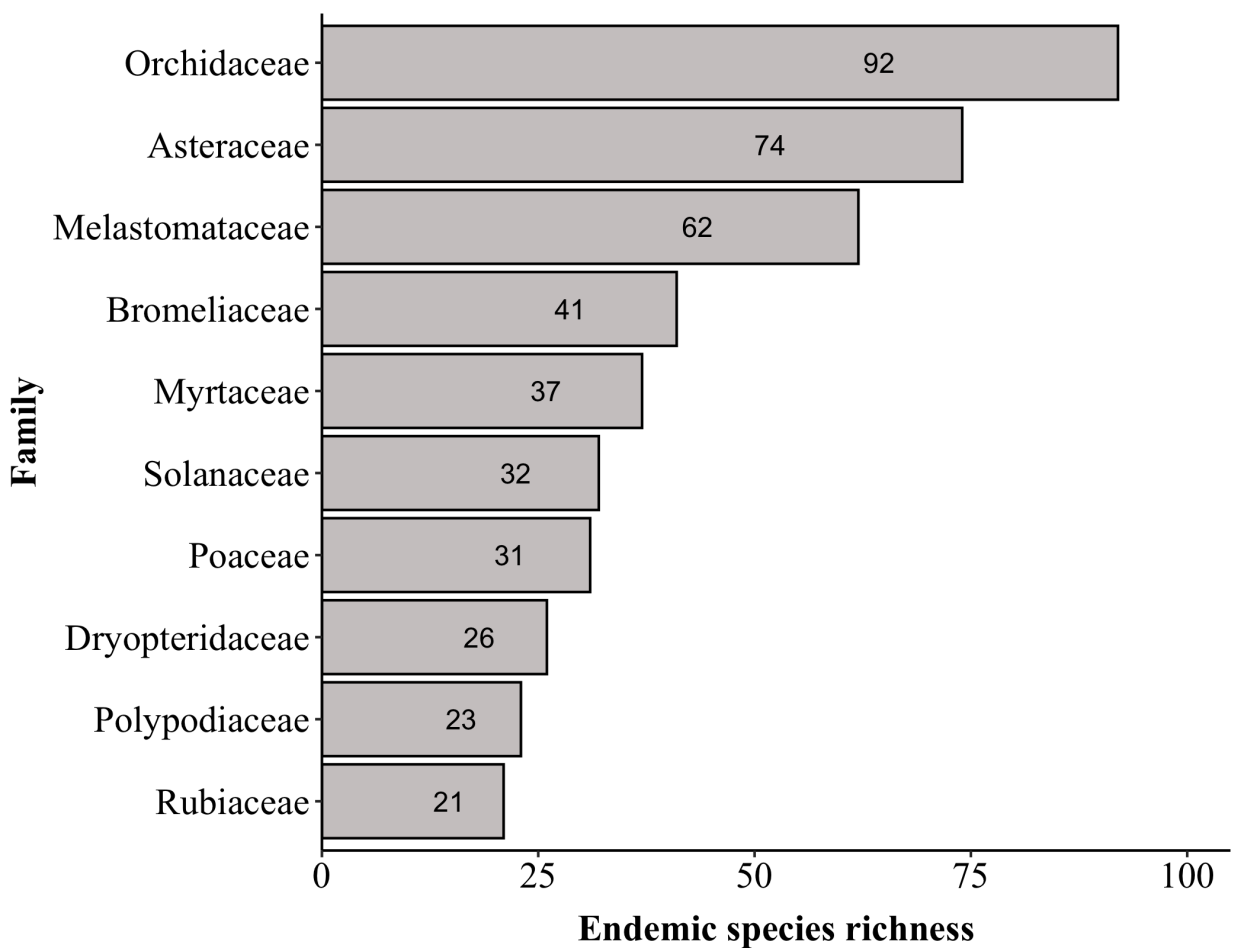
Families with the most endemic species of Brazil listed in the “Flora do Brasil 2020”, recorded in “Parque Nacional do Caparaó,” Brazil. Values inside the bars indicate the number of species.

We found a similar number of non-endemic (900) and endemic (891) species to Brazil (Fig. 7). Most of both the endemic and non-endemic species to Brazil are angiosperms (83% and 61%, respectively), followed by ferns (11% and 19%), avascular plants (4% and 18%) and lycophytes (2% and 2%). Orchidaceae and Asteraceae have the highest number of endemic species to Brazil (92 and 74, respectively), followed by Melastomataceae (62), Bromeliaceae (41) and Myrtaceae (37) (Fig. 8). The PNC protects 785 endemic species in the Atlantic Forest domain (Fig. 7), most of which are angiosperms (64.3%), followed by ferns (20.3%), avascular plants (12%) and lycophytes (3.4%). Orchidaceae has the highest number of endemic species to Atlantic Forest (67), followed by Dryopteridaceae and Asteraceae (both with 37), Melastomataceae (35), Solanaceae and Polypodiaceae (both with 33) and Bromeliaceae (32).

## Traits coverage

Within the PNC, there are 203 species considered as Least Concern (LC), 34 as Endangered (EN), 22 as Vulnerable (VU), 20 as Near Threatened (NT), seven as Critically Endangered (CR) and seven as Data Deficient (DD), based on the Brazilian National Red List - CNCFlora (http:// www.cncflora.jbrj.gov.br/portal). Most species of PNC (84%; 1,498) have not been evaluated by the Brazilian National Red List (Fig. 9; Suppl. material 1), a figure which is similar to the percentage of Brazilian species without threat evaluation (89%) (Martinelli et al. 2013).

**Figure 10.**
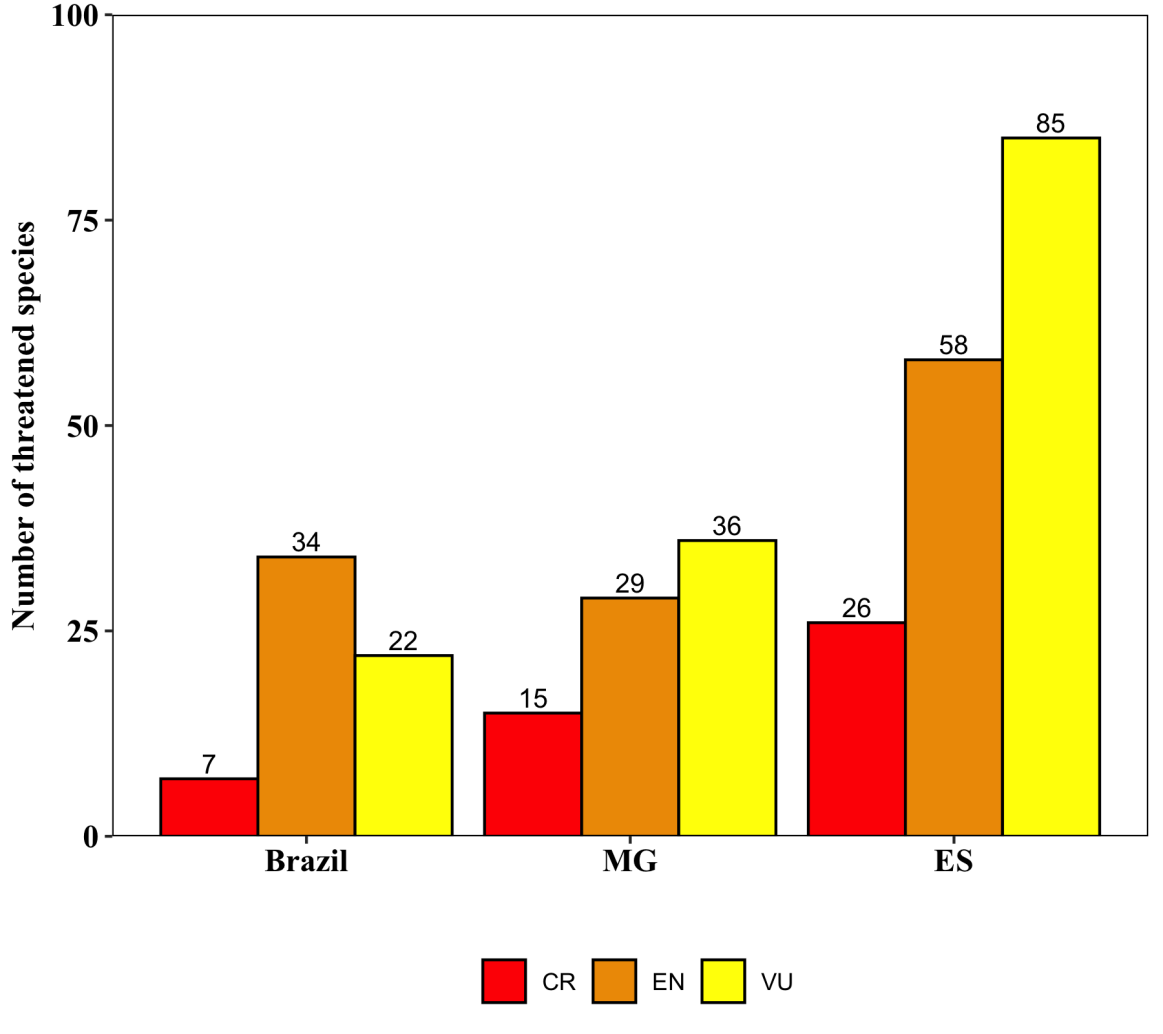
Number of threatened species housed by “Parque Nacional do Caparaó,” Brazil according to the Red List Authority for plants in Brazil - CNCFLora, Espírito Santo regional Red List (ES) and Minas Gerais regional Red List (MG). CR = Critically Endangered; EN = Endangered; VU = Vulnerable. Values above the bars indicate the number of species.

In this regard, PNC houses 63 threatened species (CR, EN and VU) of Brazilian flora and seven Data Deficient species, based on the Brazilian National Red List (Fig. 10; Table 1). These threatened (CR, EN and VU) and DD species belong to 42 families, with Orchidaceae having the highest number of threatened species (7) followed by Bromeliaceae (6), Asteraceae (5), Polypodiaceae (4) and Gesneriaceae (3) (Table 1). These three most threatened families of angiosperms of PNC are the same as those of Flora do Brasil 2020 (under construction; http://floradobrasil.jbrj.gov.br), but at different ranks (Asteraceae, Bromeliaceae and Orchidaceae) (Martinelli et al. 2013). Eight families had two threatened species and 29 families had a single threatened species (Table 1).

According to regional Red Lists, the PNC houses 169 threatened species of ES (Fraga et al. 2019; Fig. 10) and 80 threatened species of MG (COPAM-MG 2008), representing 12% of the threatened flora of ES (1,430 threatened species of ES; Suppl. material 1) and 7% of the threatened flora of MG (1,124 threatened species of MG; Suppl. material 1).

Considering the entire flora of PNC, 24 species belonging to 18 families are rare in Brazil. Asteraceae and Orobanchaceae have the highest number of rare species (three) followed by Bromeliaceae and Scrophulariaceae (two each, Table 2). Fourteen families have a single rare species (Table 2). We retrieved five species, four angiosperms and one fern, which are priorities for conservation in PNC (Table 1).

## Usage licence

### Usage licence

Open Data Commons Attribution License

## Data resources

### Data package title

A list of land plants of Parque Nacional do Caparaó, Brazil, highlights the presence of sampling gaps within this protected area.

### Resource link

https://ckan.jbrj.gov.br/dataset/2020_caparao_list

### Number of data sets

1

### Data set 1.

#### Data set name

List of species of the Parque Nacional do Caparaó.

#### Number of columns

21

#### Download URL

https://ckan.jbrj.gov.br/dataset/d8886b0b-f47d-4e2c-aac3-f68d5f1e3036/resource/b184ef97-c1ca-463f-9f35-e7195717b74a/download/table-s1.csv

#### 

**Data set 1.** 

Column label	Column description
Groups	Controlled vocabulary ("Angiosperms", "Ferns", "Hornworts, "Liverworts", "Lycophytes", "Mosses")
Families	The full scientific name of the family in which the taxon is classified
Genera	The full scientific name of the genus in which the taxon is classified.
Species	The full scientific name
Author name	Authorship of the scientific name
Number of specimens	Number of specimens for the species
Database or herbarium code	Botanical collection or database of origin of the record
Barcode	The unique identifier for the record within botanical collections
catalogue.number or collector name and number	Field sed in case of the absence of the barcode
The species present a single record?	Controlled vocabulary ("yes", "no")
The species present only old records?	Controlled vocabulary ("yes", "no", "NA")
The species is native to Brazil?	Controlled vocabulary ("native", "non-native")
The species is endemic to Brazil?	Controlled vocabulary ("endemic", "non-endemic")
The species occur in the Atlantic Forest, according to Flora do Brasil 2020?	Controlled vocabulary ("yes", "no", "no information")
Phytogeographic domain obtained by R	Brazilian phytogeographic domains where the species occur
The species is endemic to Atlantic Forest?	Controlled vocabulary ("endemic", "non-endemic", "no information")
The species occur in ES and MG States according to the Flora do Brasil 2020?	Controlled vocabulary including the Brazilian states codes ("MG", "ES", "ES and MG", "no", "no information")
Threat category according to CNCFLORA	Followed the Red List Authority for plants in Brazil - Centro Nacional de Conservação da Flora - CNCFlora (http://www.cncflora.jbrj.gov.br/portal); DD = Data deficient, NT = Near Threatened, VU = Vulnerable, CR = Critically Endangered, EN = Endangered, LC = Least Concern, NE = Not evaluated
Threat category (CR, EN or VU) according to the lists of Espírito Santo State	Followed the regional Red List of the Espírito Santo State
Threat category (CR, EN or VU) according to the lists of Minas Gerais State	Followed the regional Red List of the Minas Gerais State
The species occur in Dutra et al. 2015 list?	Compared with Dutra el al. (2015) <https://doi.org/10.1590/2175-7860201566414>

## Additional information

### Conclusions and prospects

**Figure 11.**
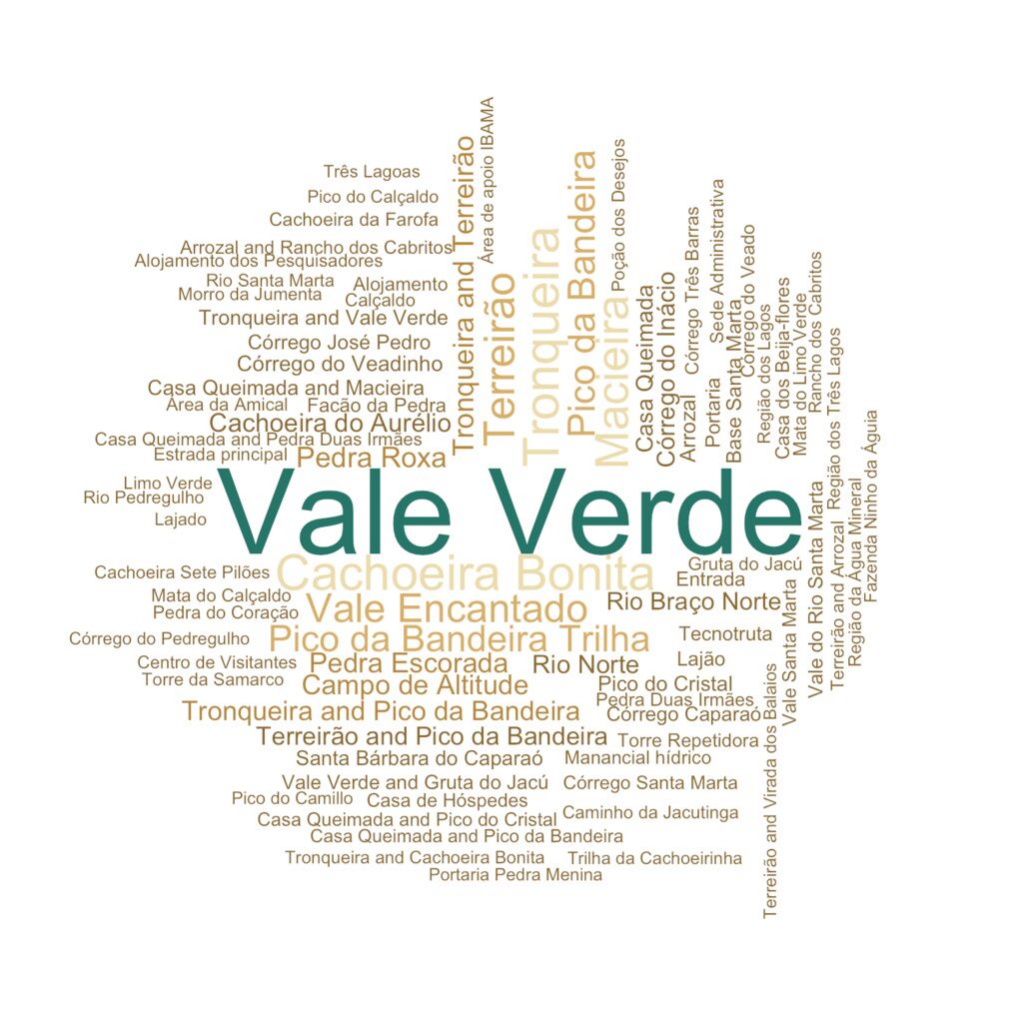
Word cloud of locations where plants of “Parque Nacional do Caparaó,” Brazil, were collected. The different colours and sizes of the letters represent the frequency of collections at the localities within the Park; larger font size represents a greater number of collections.

The analysis of specimens collected in the PNC allowed us to detect a spatial collection gap. Most (79%) of the specimens analysed were collected in a small portion of the Park (21% of the area of the Park) located in MG. These collections were mainly made in easily accessible places of PNC, such as Vale Verde, Cachoeira Bonita, Vale Encantado, Tronqueira and Terreirão (Fig. 11). Collection bias towards access routes is a common sampling problem that has been reported for various taxonomic groups (i.e. vertebrates, invertebrates and angiosperms) throughout all Brazilian phytogeographic domains and can affect the detection of spatial patterns of species diversity (Oliveira et al. 2016).

**Figure 12.**
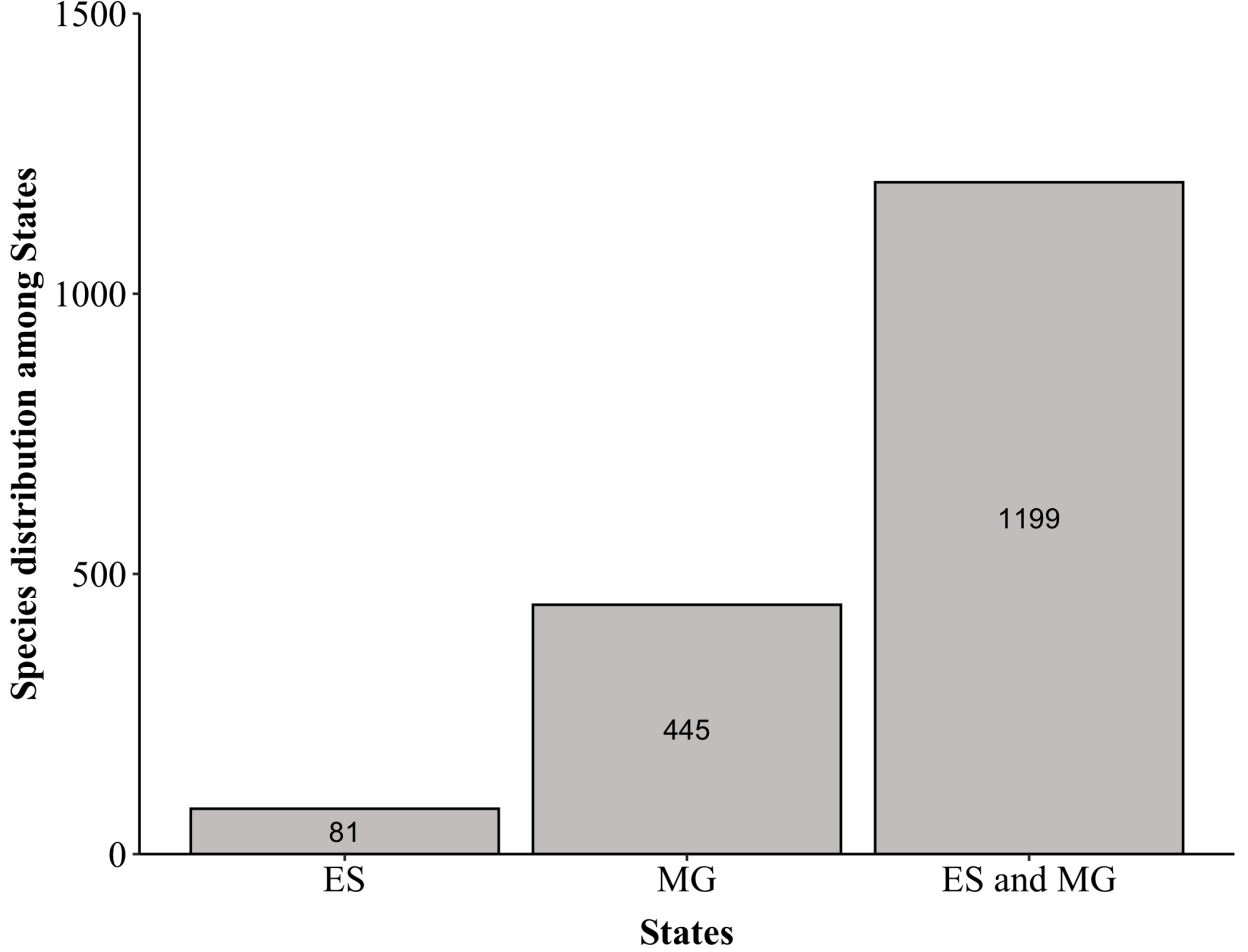
Species of “Parque Nacional do Caparaó,” Brazil, distributed betweenthe States of Espírito Santo (ES) and Minas Gerais (MG) according to Flora do Brasil (2020). Values inside the bars indicate the number of species.

Furthermore, amongst the species found in PNC, 445 are described by Flora do Brasil under construction (2020) as occurring in MG, while only 81 species are described as occurring in ES, whereas a total of 1,199 occur in both States (ES and MG; Fig. 12 and Suppl. material 1). The Park also houses only 14% (873 species) of the species, 29% (398) of the genera and 61% (110) of the families of angiosperms listed for ES (Suppl. material 1; Dutra et al. 2015). We did not find information about the state of occurrence for 13 species and 53 species did not appear in Flora do Brasil under construction (2020) as occurring in either ES or MG (Fig. 12 and Suppl. material 1). Despite the Atlantic Forest being one of the most sampled phytogeographic domains in Brazil (Oliveira et al. 2019), our results demonstrate that even areas that have been extensively studied can have collection gaps. Thus, deeper knowledge of the flora of PNC requires increased collection effort in the less accessible areas of the Park located mainly in ES.

A particularly interesting result was the 53 species in the list of PNC that were not recorded as occurring in ES or MG. It may be that these species are new occurrences for MG or ES. One recent study, involving the family Araceae in PNC, reported *Xanthosoma maximilianii* Schott and *Philodendron acutatum* Schott of these 53 species, as new occurrences for ES and MG, respectively (Camelo et al. 2020), emphasising the importance of more in-depth assessments of the occurrence of the other 51 species.

The species list for PNC, presented here, was prepared using information acquired from online databases and validated by taxonomists. This method is considered good practice for estimating species diversity in the “era of big data” (Maldonado et al. 2015). The retrieval of information contained in the online databases revealed that PNC houses 10% (1,699 species) of the species recognised for the Brazilian Atlantic Forest (i.e. 17,776 species; Flora do Brasil under construction 2020) and 8% (785) of the species endemic to this domain (i.e. 10,024 species; Flora do Brasil under construction 2020), with 6% (505) of angiosperms, 31% (186) of lycophytes and ferns and 14% (94) of avascular plants. In conclusion, the species list of PNC allowed us not only to identify the species that occur in this protected area, but also to identify gaps in knowledge that can help direct the allocation of future collecting efforts.

## Supplementary Material

EFF1E663-1274-5034-8A19-3D0953A3236B 10.3897/BDJ.8.e59664.suppl1 Supplementary material 1 List of plants occurring in the "Parque Nacional do Caparaó" providing information on the number of specimens per species in the database, presence of single records, presence of old records, origin (native vs. non-native), endemism in Brazil, species occurrence in the Atlantic Forest, Phytogeographic domain, endemism in Atlantic Forest, Threat category according to CNCFLORA, Threat category (CR, EN or VU) according to the lists of Espírito State, Threat category (CR, EN or VU) according to the lists of Minas Gerais State and species occurrence in the list of Dutra et al. 2015. No information = indicates that data is lacking for that species. Data type Inventory regional File: oo_462550.txt https://binary.pensoft.net/file/462550 Moreira et al.

## Figures and Tables

**Figure 2a.**
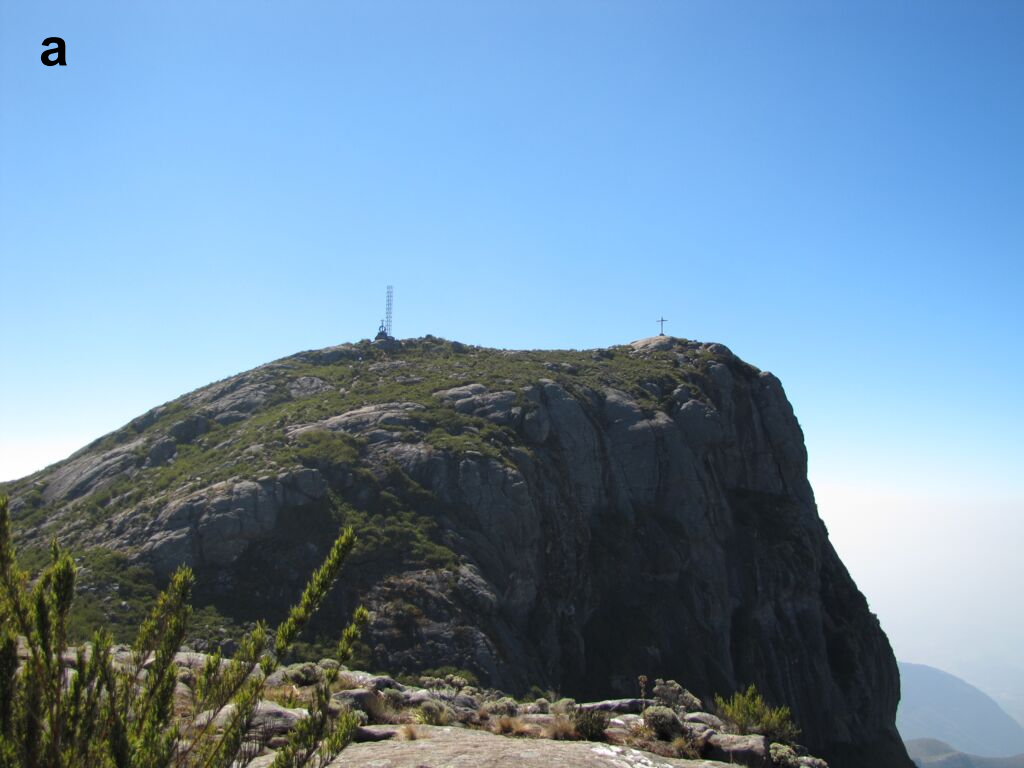
High-altitude grassland, “Pico da Bandeira”

**Figure 2b.**
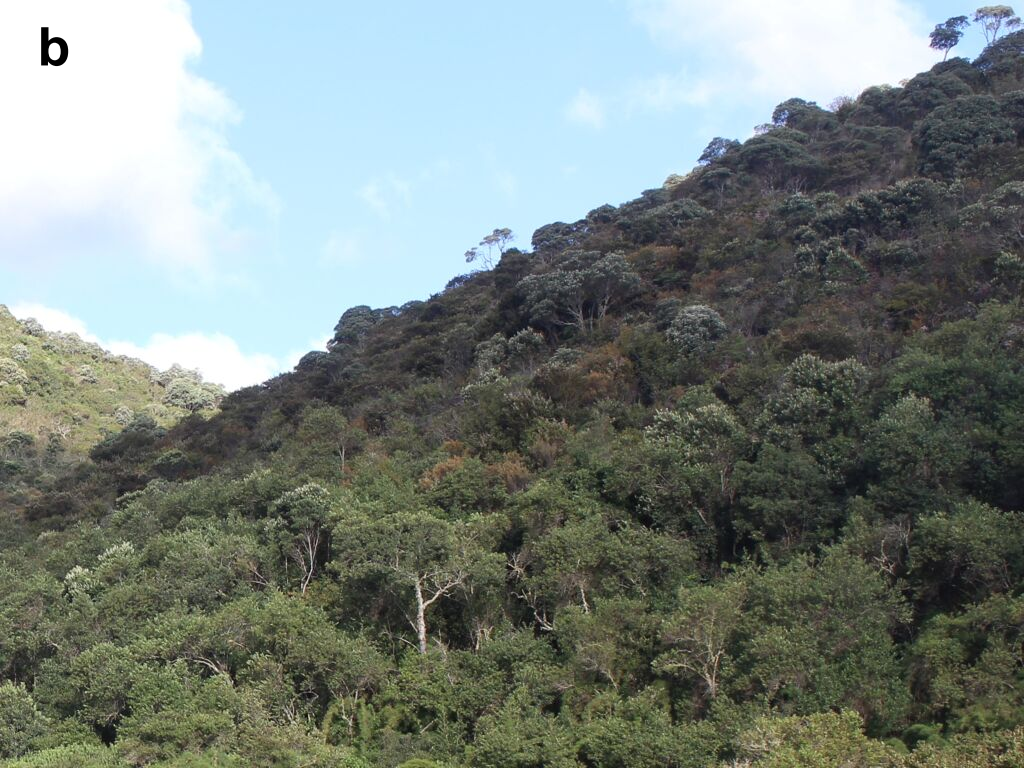
Ombrophilous forest

**Figure 2c.**
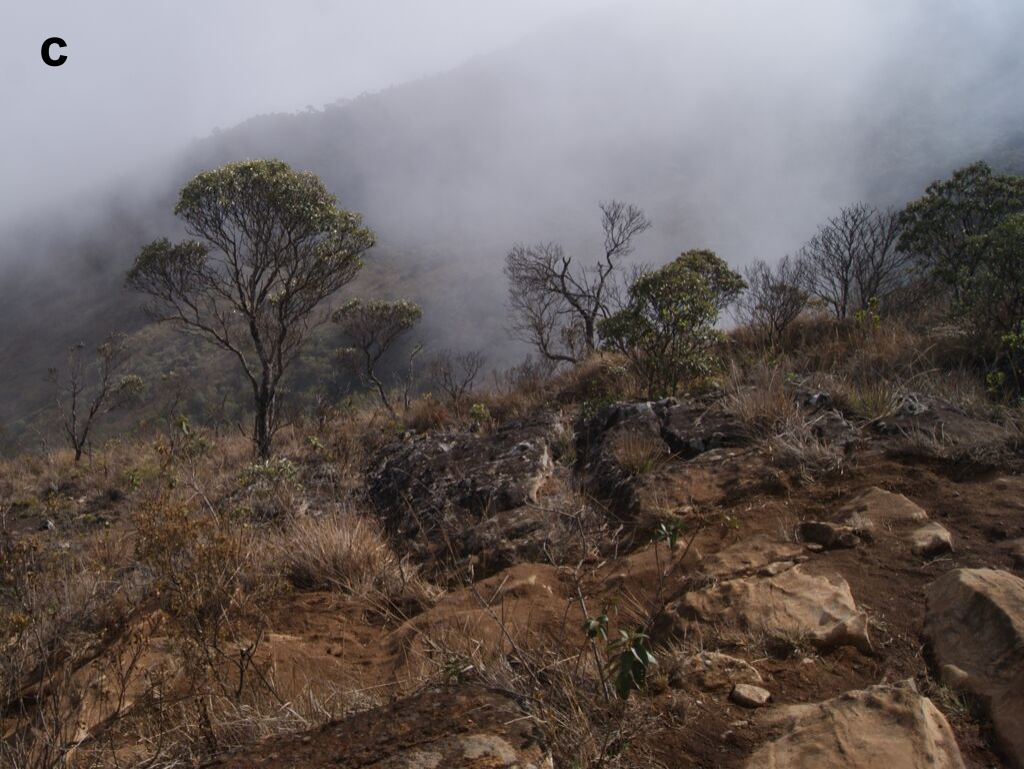
High-altitude grassland

**Figure 2d.**
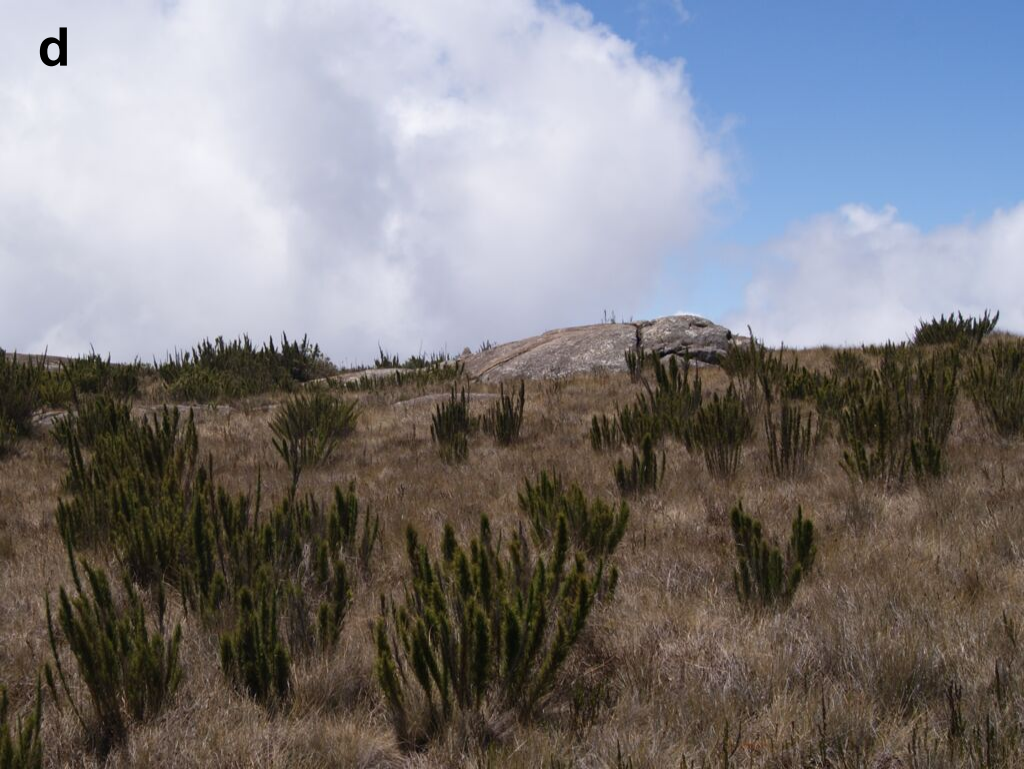
High-altitude grassland dominated by *Chusquea* Kunth (Poaceae) vegetation

**Figure 3a.**
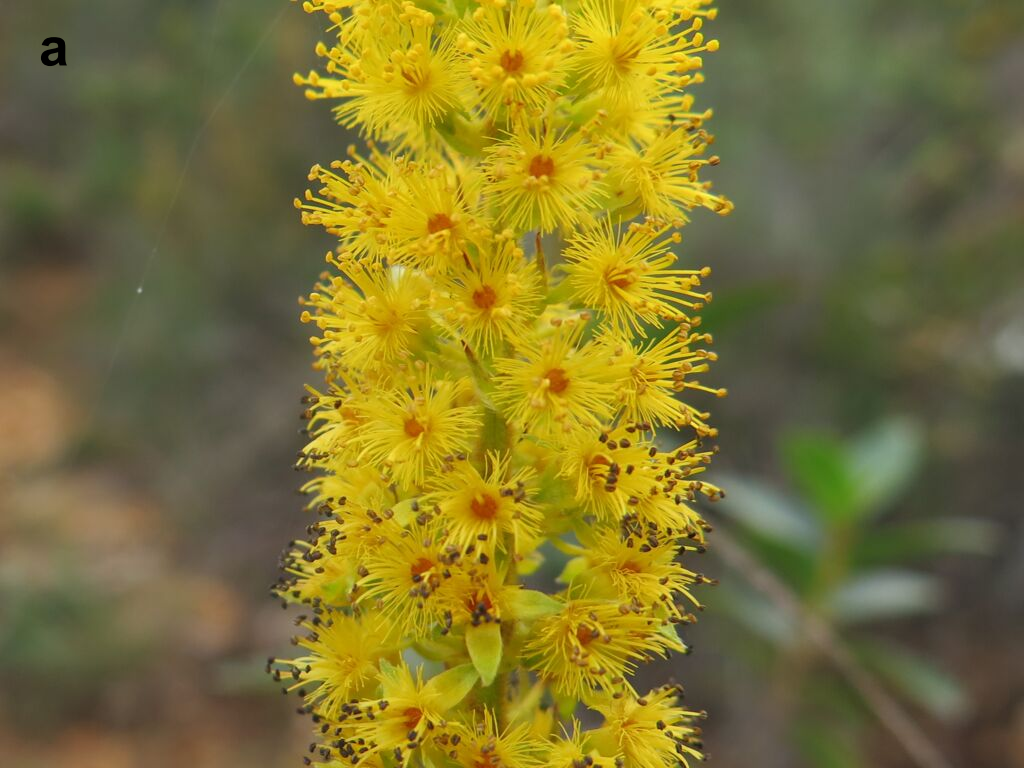
*Abatia americana* (Gardner) Eichler - Salicaceae

**Figure 3b.**
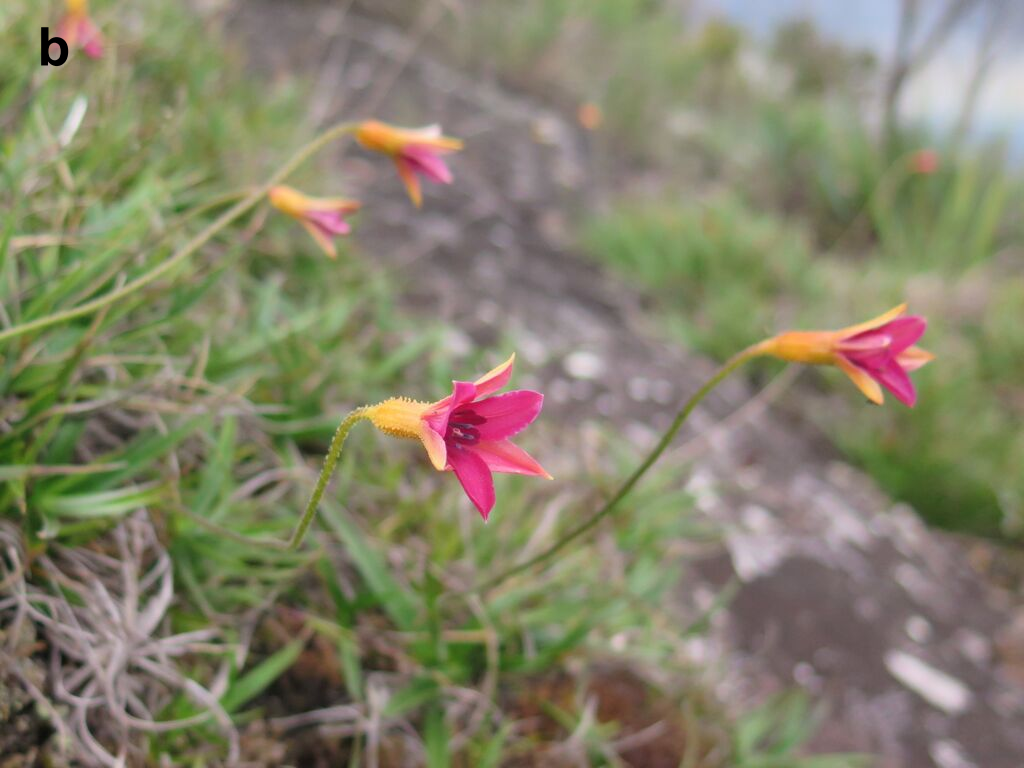
*Barbacenia irwiniana* L.B.Sm. - Velloziaceae

**Figure 3c.**
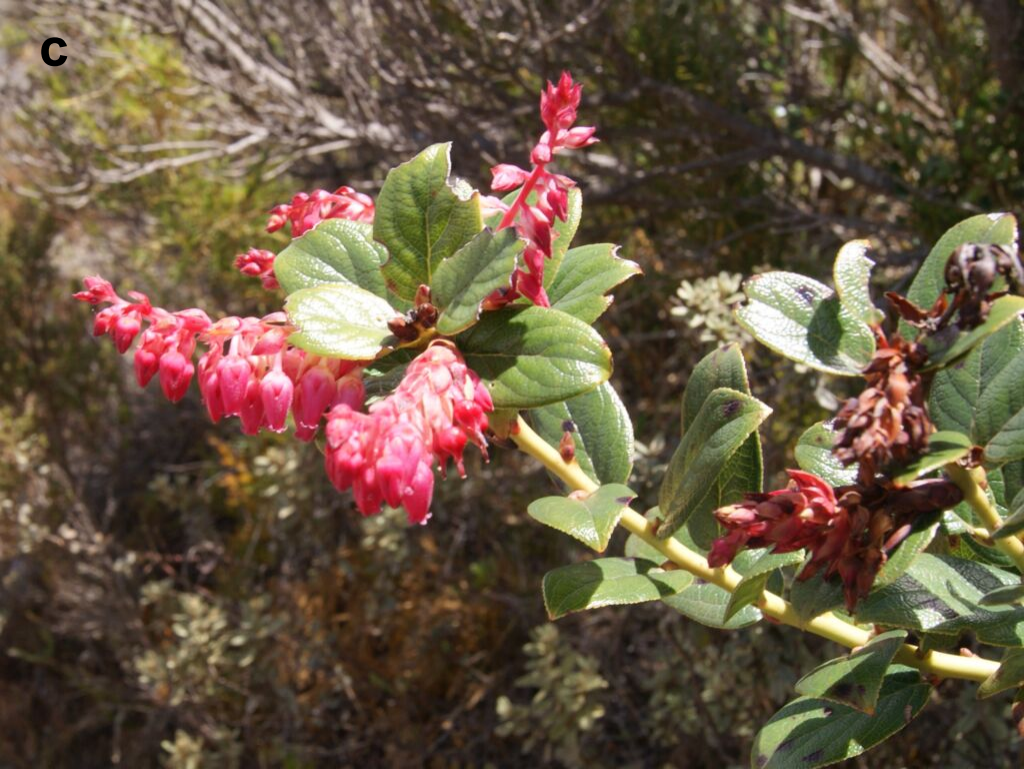
*Gaultheria serrata* (Vell.) Sleumer ex Kin.-Gouv. - Ericaceae

**Figure 3d.**
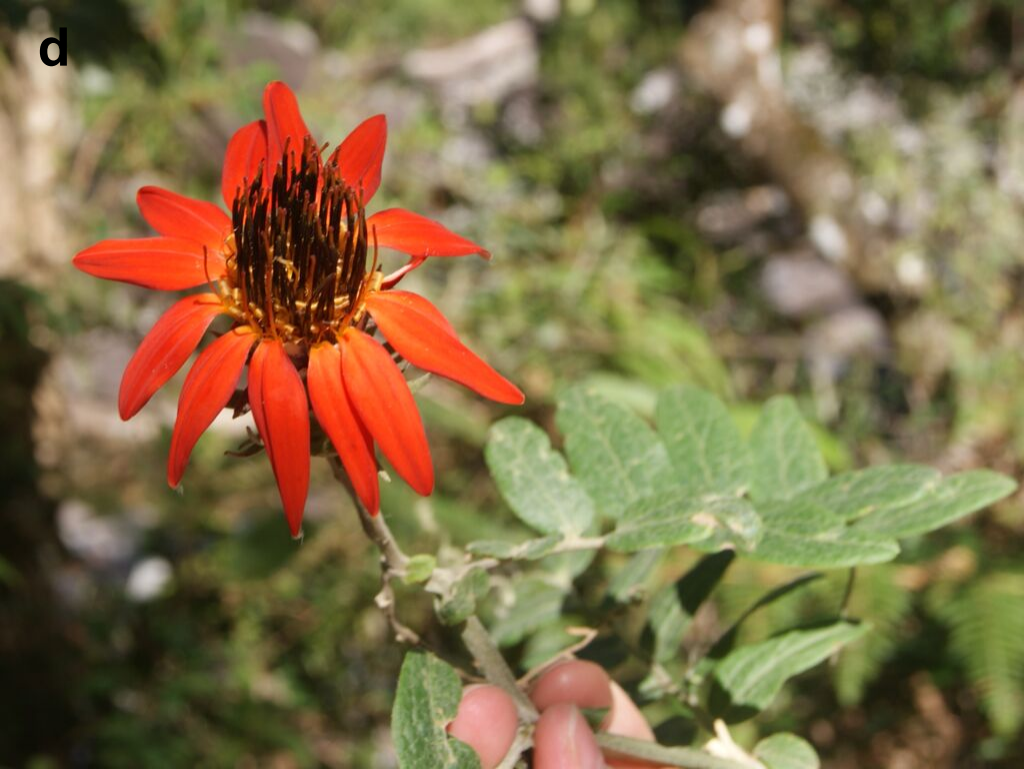
*Mutisia campanulata* Less. - Asteraceae

**Figure 3e.**
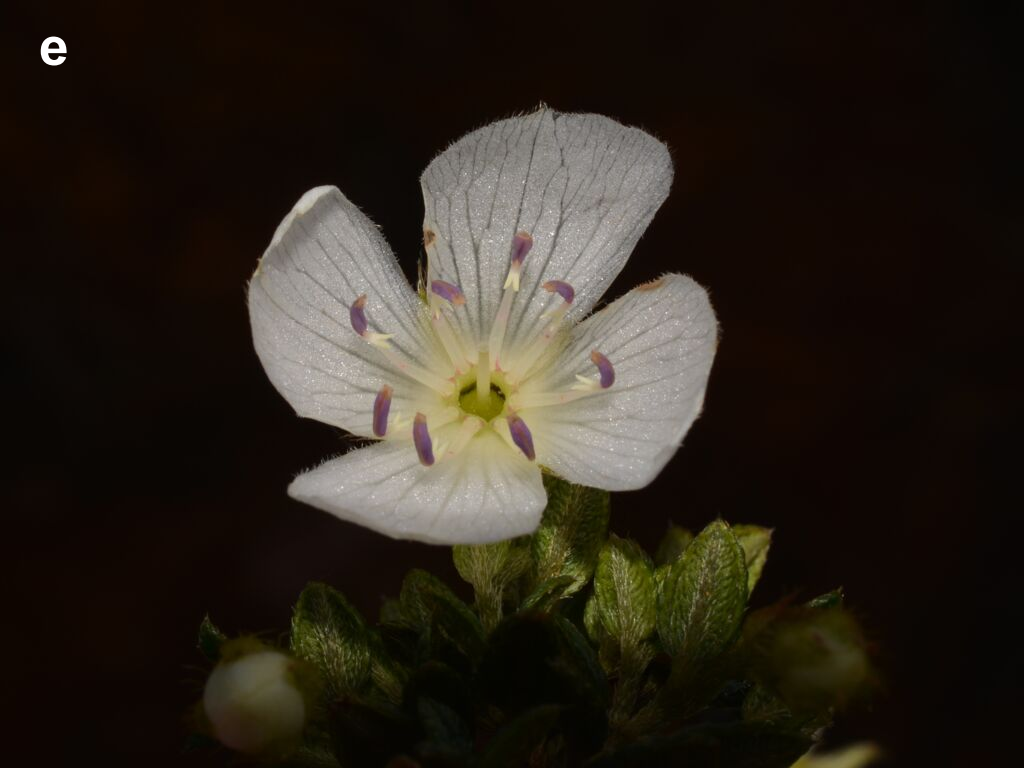
*Pleroma microphyllum* (Cogn.) P.J.F.Guim. & Michelang. - Melastomataceae

**Figure 3f.**
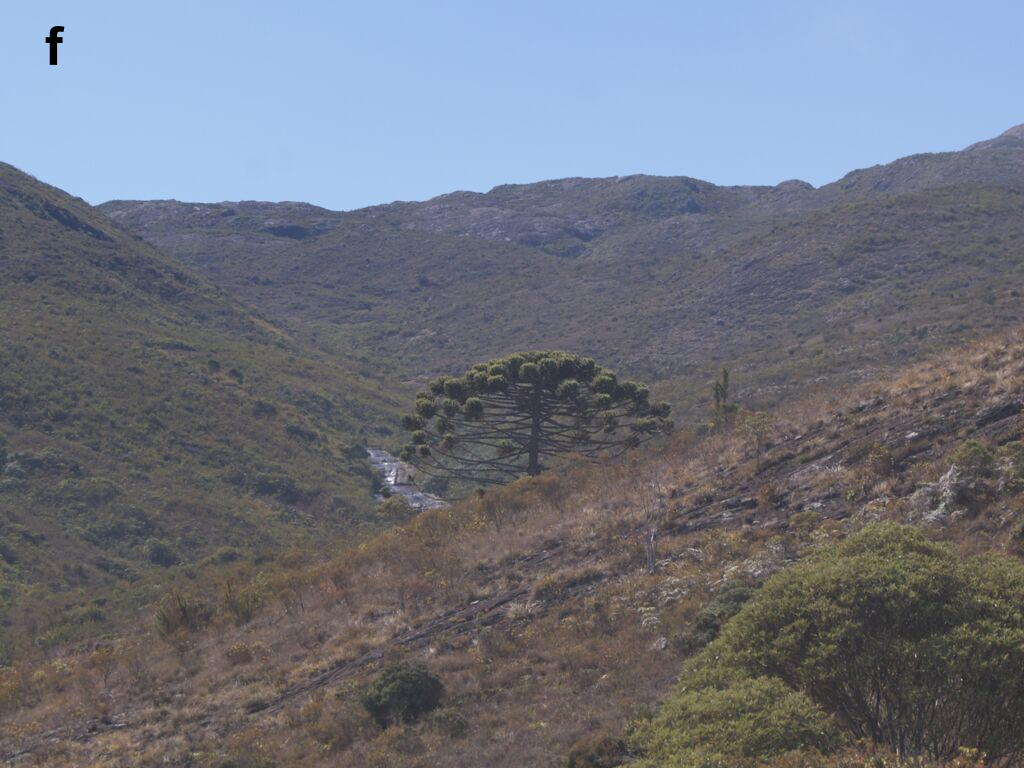
*Araucaria angustifolia* (Bertol.) Kuntze - Araucariaceae

**Figure 4a.**
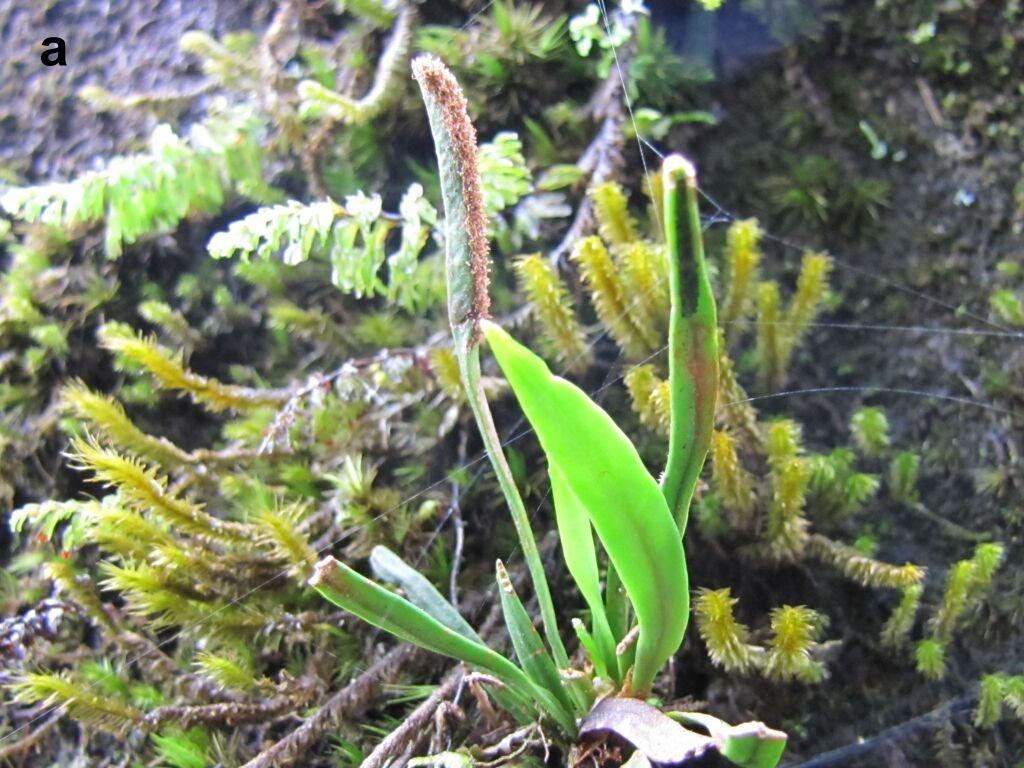
*Cochlidium punctatum* (Raddi) L.E.Bishop - Polypodiaceae

**Figure 4b.**
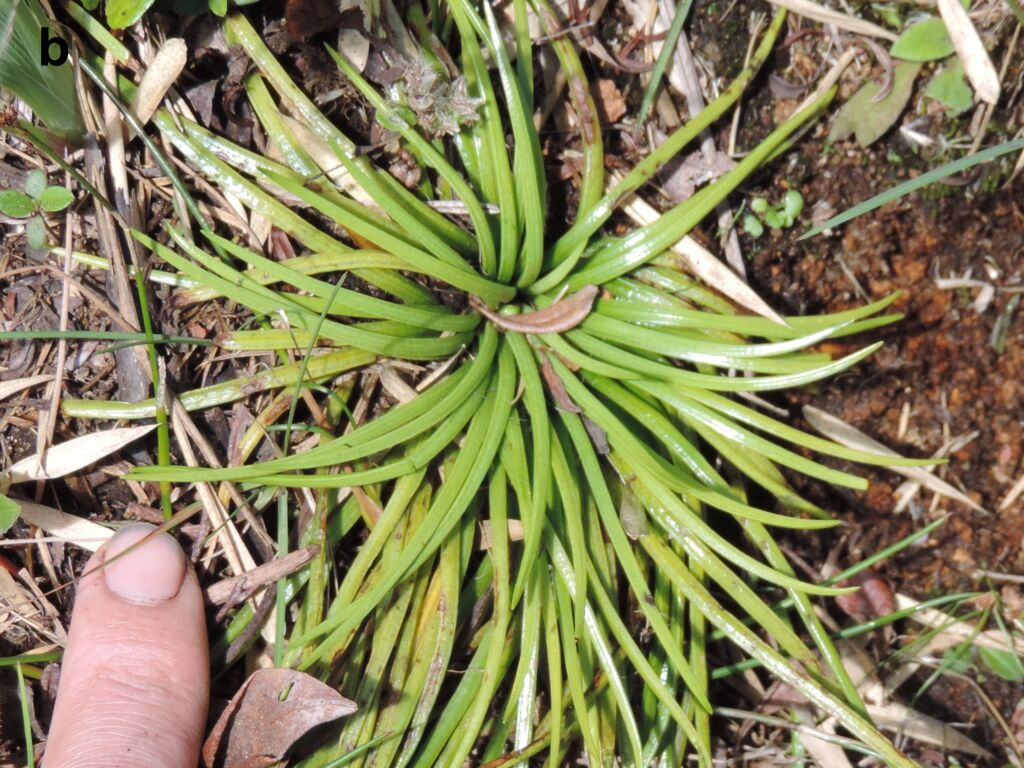
*Isoetes caparaoensis* J.B.S.Pereira - Isoetaceae

**Figure 4c.**
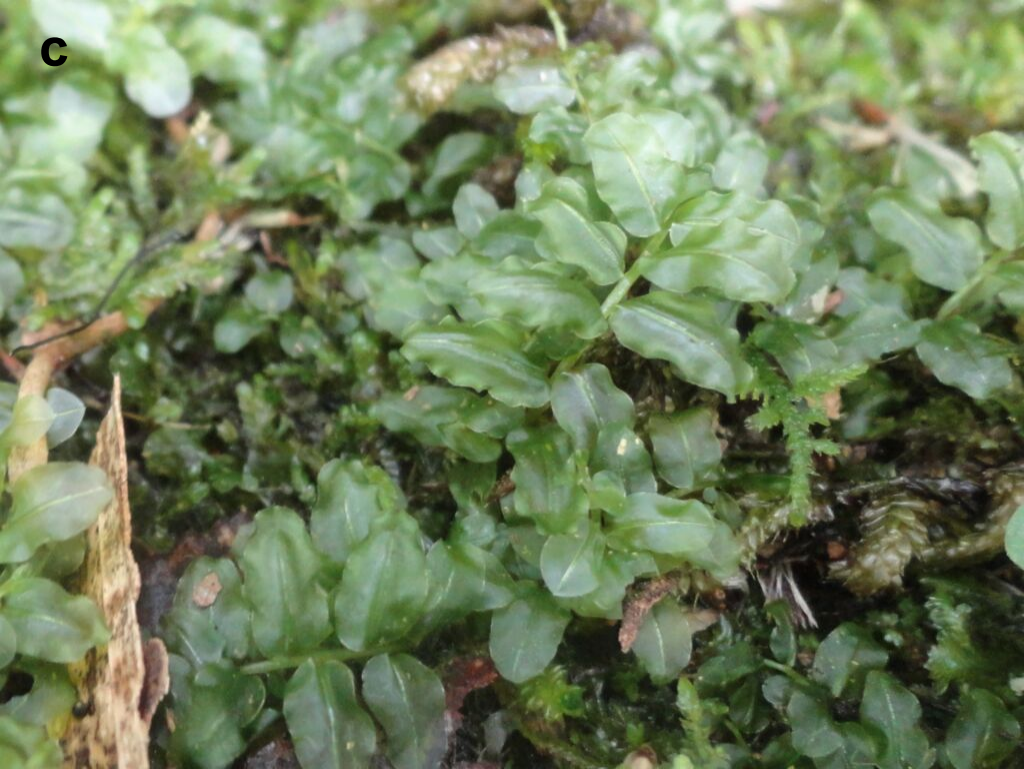
*Plagiomnium rhynchophorum* (Hook.) T.J.Kop. - Mniaceae

**Figure 4d.**
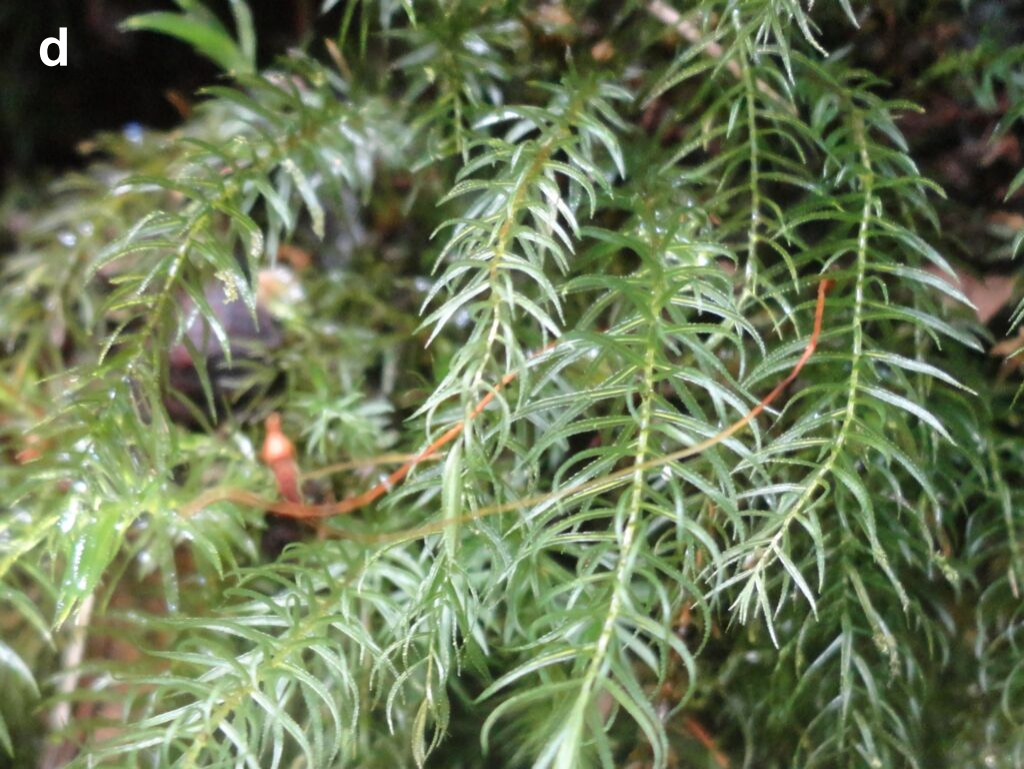
*Pyrrhobryum spiniforme* (Hedw.) Mitt. - Rhizogoniaceae

**Figure 5a.**
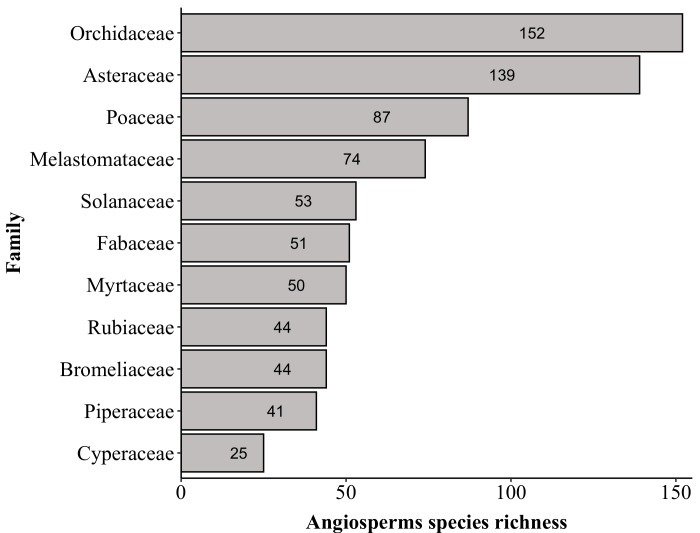
Top ten families of angiosperms

**Figure 5b.**
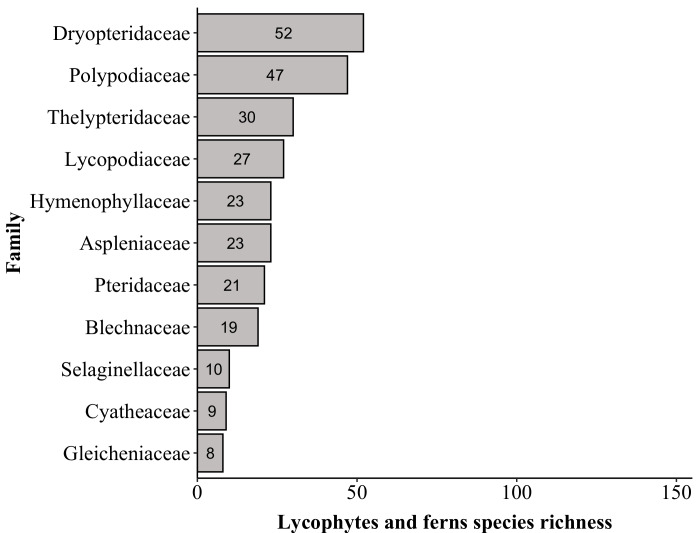
Top ten families of lycophytes and ferns

**Figure 5c.**
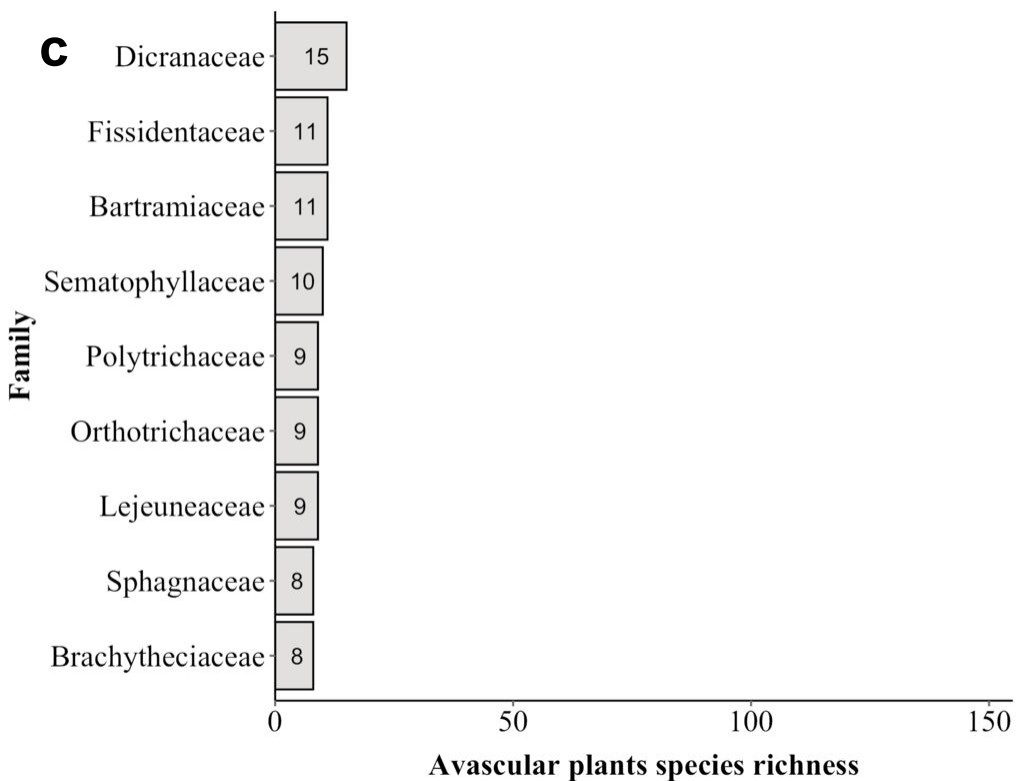
Top five families of avascular plants

**Figure 5d.**
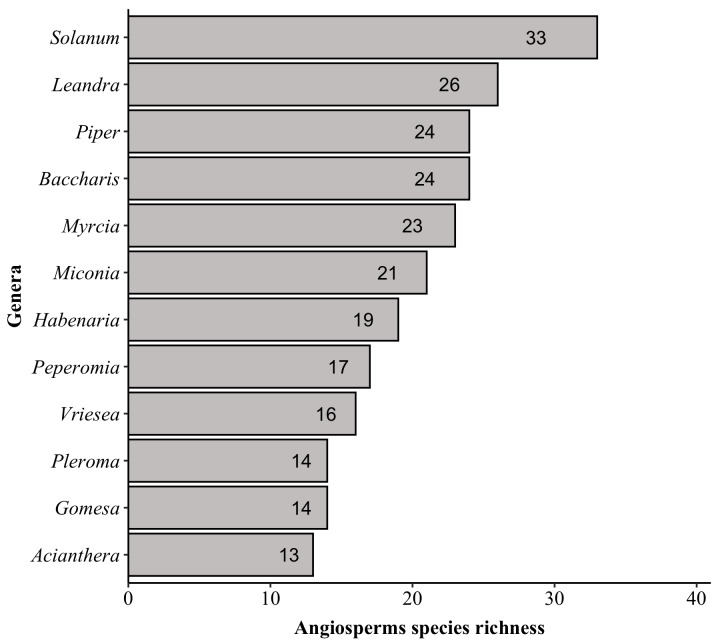
Top ten genera of angiosperms

**Figure 5e.**
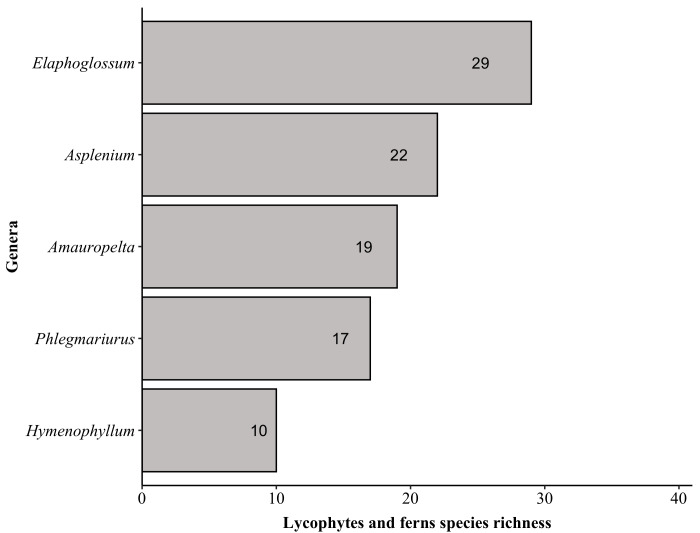
Top five genera of lycophytes and ferns

**Figure 5f.**
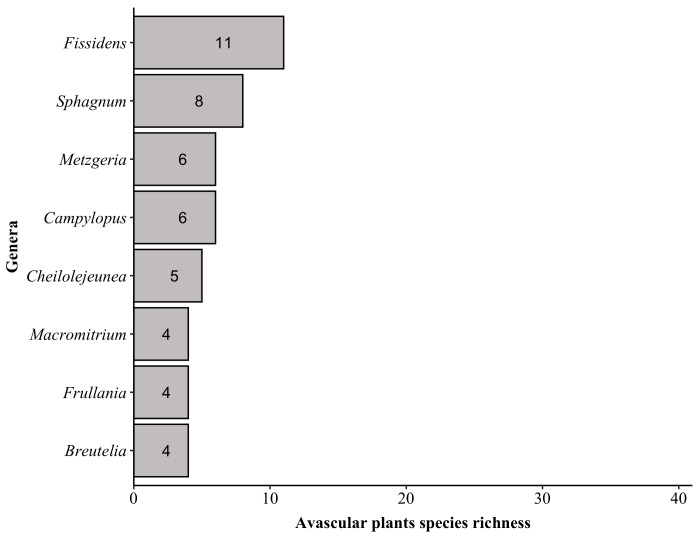
Top five genera of avascular plants

**Figure 9a.**
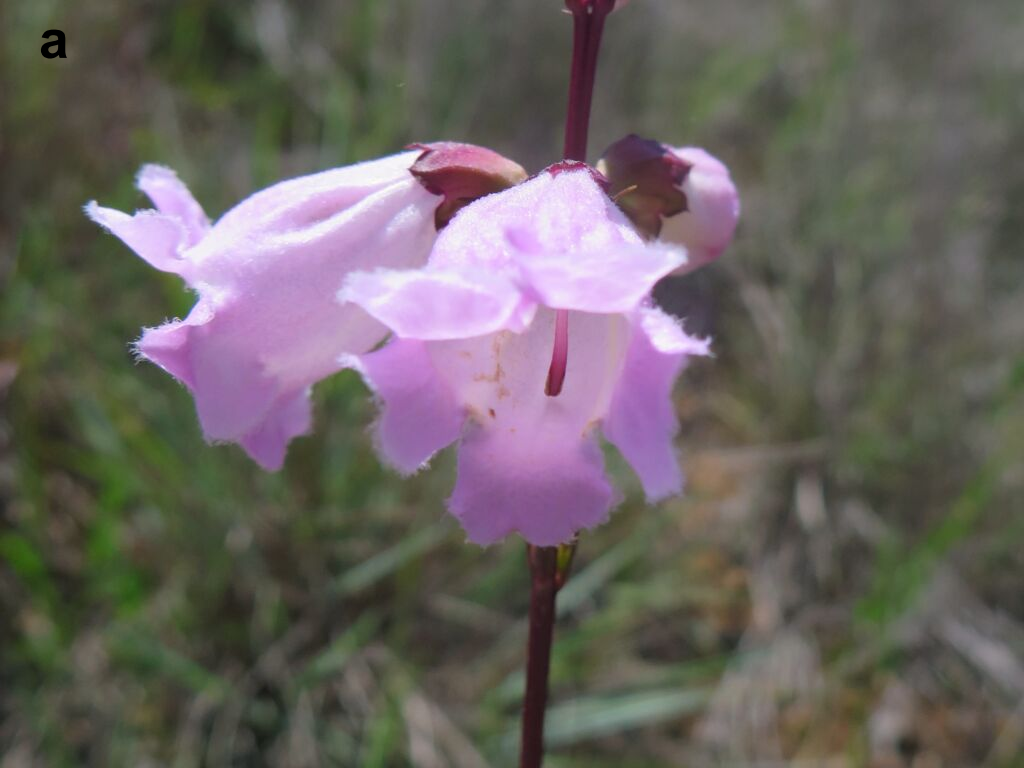
*Agalinis bandeirensis* Barringer - Orobanchaceae (CR)

**Figure 9b.**
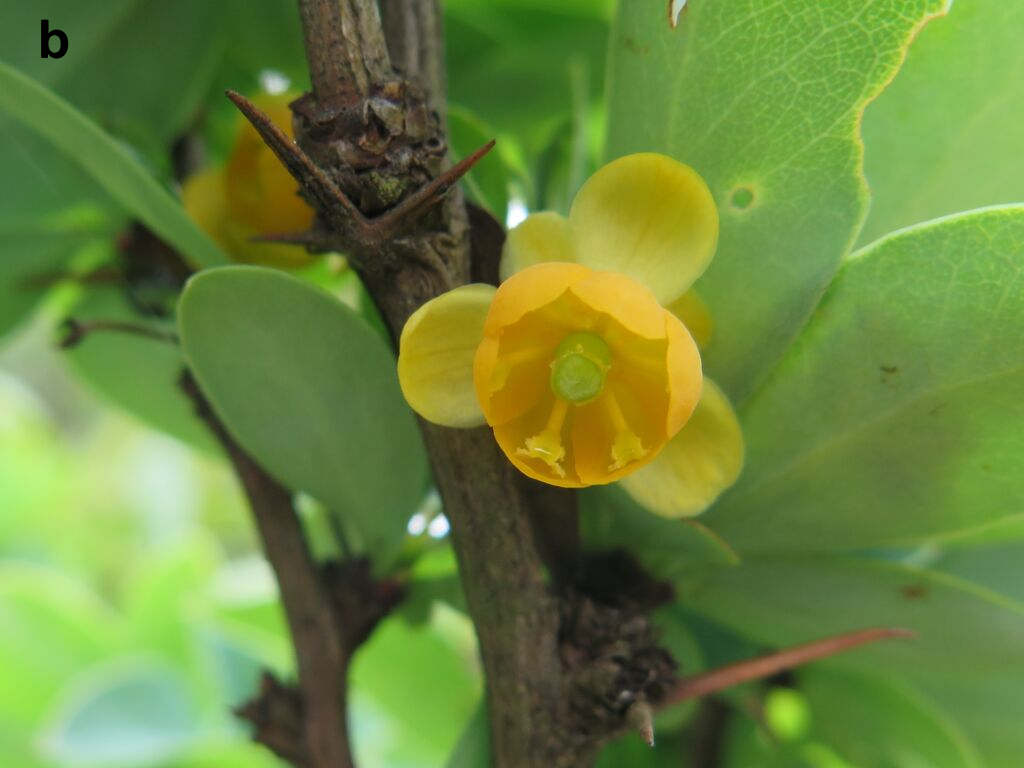
*Berberis campos-portoi* Brade - Berberidaceae (CR)

**Figure 9c.**
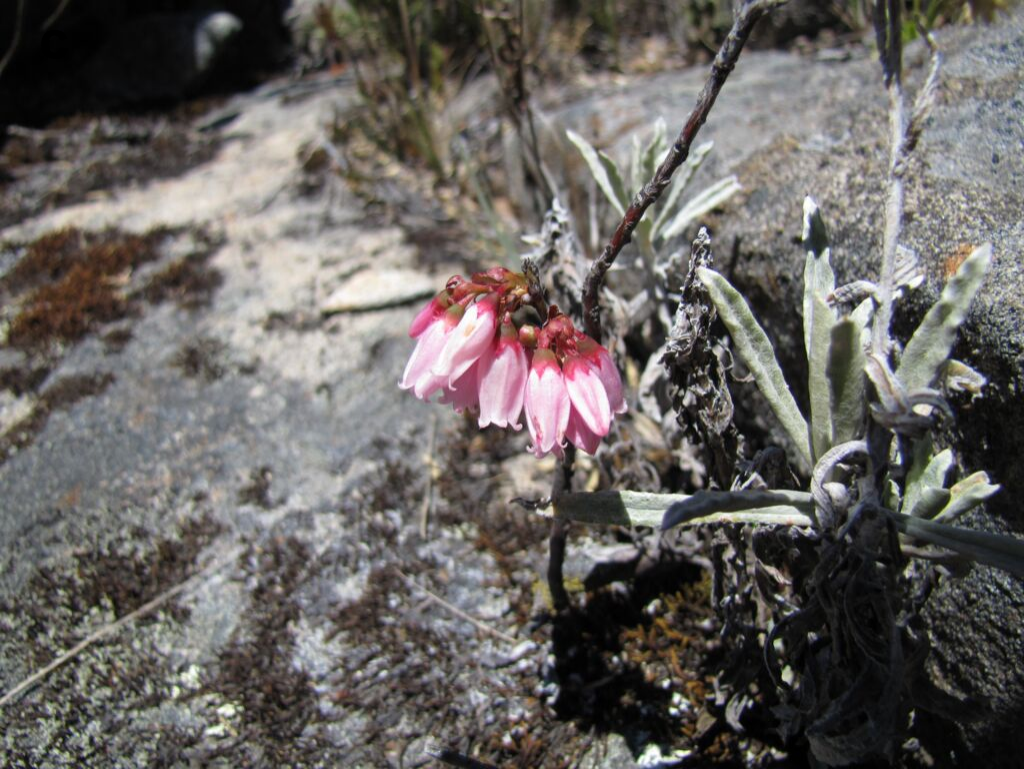
*Gaylussacia caparoensis* Sleumer - Ericaceae (EN)

**Figure 9d.**
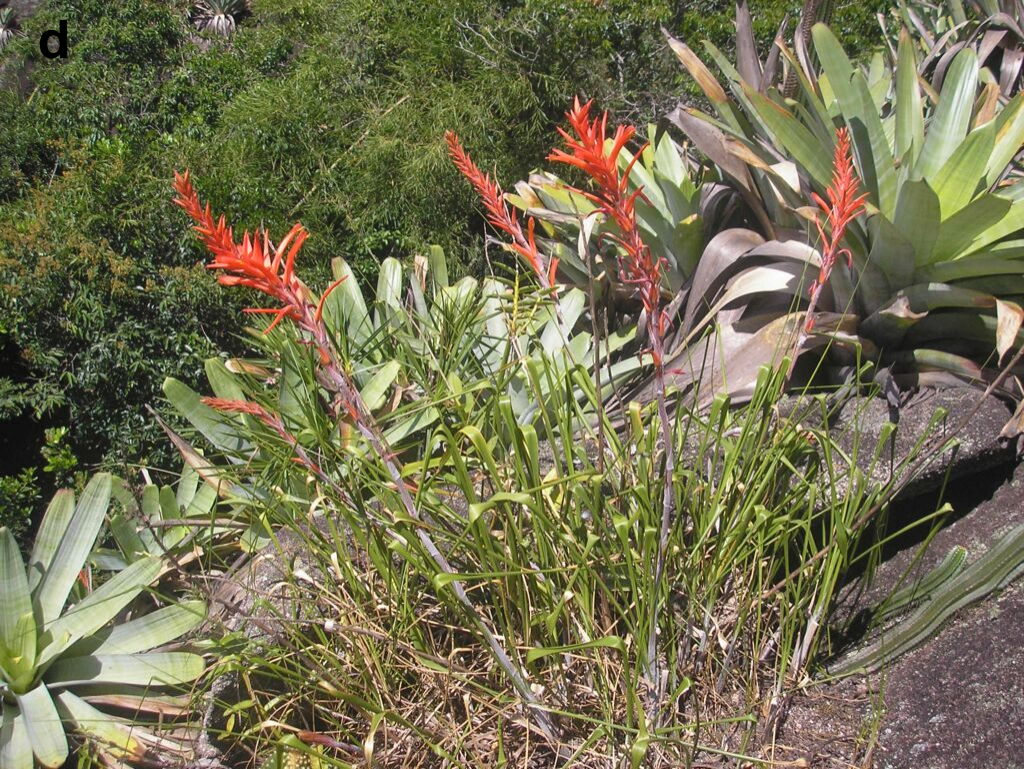
*Pitcairnia decidua* L.B.Sm. - Bromeliaceae (EN)

**Figure 9e.**
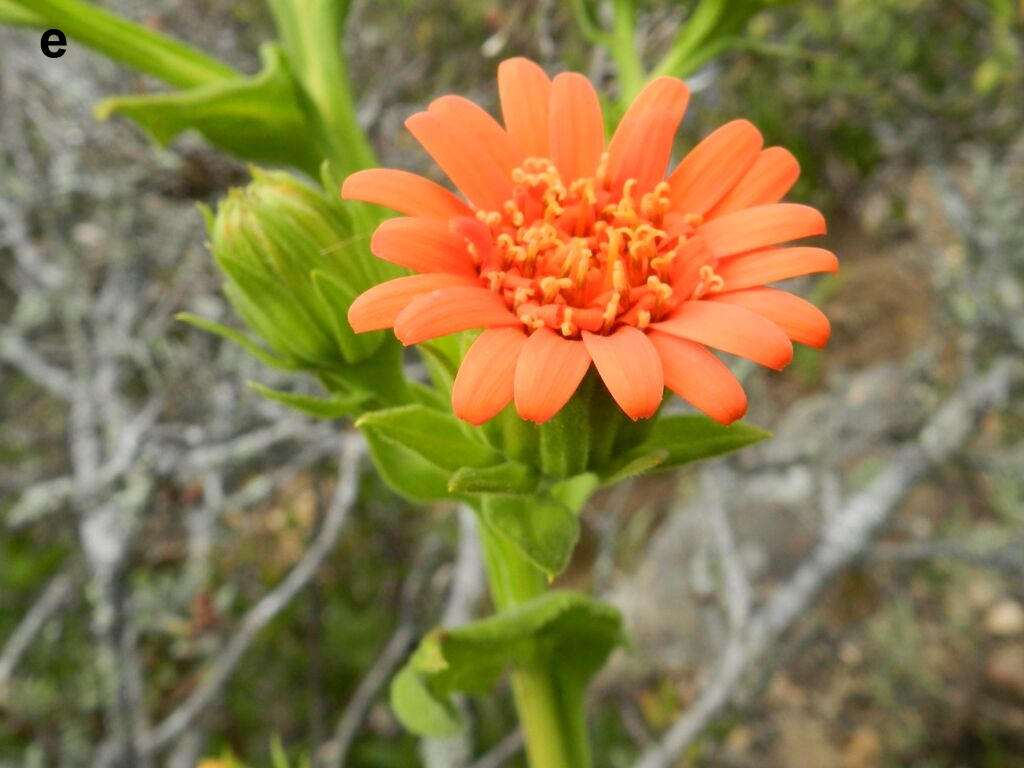
*Trixis glaziovii* Baker - Asteraceae (VU)

**Figure 9f.**
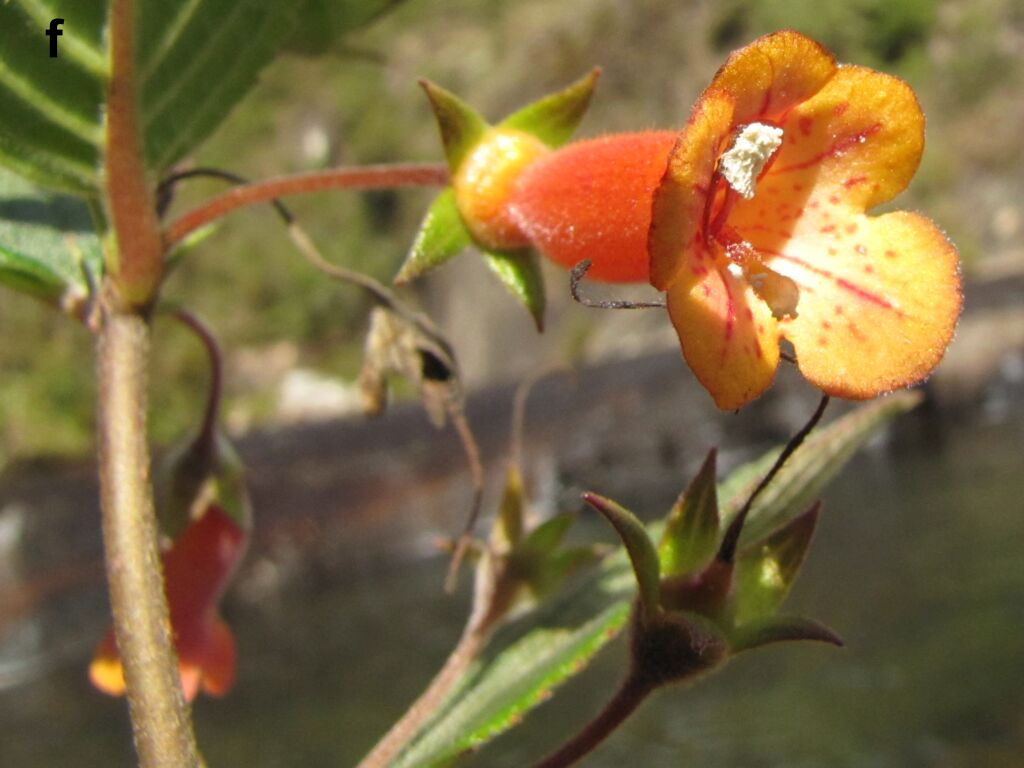
*Vanhouttea leonii* Chautems - Gesneriaceae (EN)

**Table 1.** Threatened and Data Deficient species of plants occurring in “Parque Nacional do Caparaó,” Brazil, their respective groups, families and threat category according to the Red List Authority for plants in Brazil - CNCFLora (CR = Critically Endangered, VU = Vulnerable, EN = Endangered and DD = Data Deficient). *Priority species for conservation.

**Species**	**Red list category**
**Angiosperms**	
** Amaryllidaceae **	
*Hippeastrum morelianum* Lem.	VU
** Apiaceae **	
*Eryngium glaziovianum* Urb.	DD
** Apocynaceae **	
*Ditassa leonii* Fontella & T.U.P.Konno	VU
*Oxypetalum leonii* Fontella	EN
** Araliaceae **	
*Hydrocotyle langsdorffii* DC.	EN
** Arecaceae **	
*Euterpe edulis* Mart.	VU
** Asteraceae **	
*Chionolaena lychnophorioides* Sch.Bip.	VU
*Mikania additicia* B.L.Rob.	EN
*Mikania hastato-cordata Malme	VU
*Senecio caparaoensis* Cabrera	EN
*Trixis glaziovii* Baker	VU
** Berberidaceae **	
*Berberis campos-portoi* Brade	CR
** Bromeliaceae **	
*Aechmea vanhoutteana* (Van Houtte) Mez	VU
*Neoregelia brownii* Leme	CR
*Neoregelia ruschii* Leme & B.R.Silva	EN
*Pitcairnia decidua* L.B.Sm.	EN
*Quesnelia kautskyi* C.M.Vieira	VU
*Vriesea pabstii* McWill. & L.B.Sm	DD
** Burmanniaceae **	
*Burmannia aprica* (Malme) Jonker	DD
** Cactaceae **	
*Schlumbergera kautskyi* (Horobin & McMillan) *N.P.Taylor*	EN
*Schlumbergera microsphaerica* (K.Schum.) Hoevel	VU
** Clusiaceae **	
*Clusia aemygdioi* Gomes da Silva & B.Weinberg	EN
** Ericaceae **	
*Gaylussacia caparoensis* Sleumer	EN
** Fabaceae **	
*Bionia bella* Mart. ex Benth.	DD
** Gentianaceae **	
*Senaea janeirensis* Brade	EN
** Gesneriaceae **	
*Codonanthe carnosa* (Gardner) Hanst.	VU
*Vanhouttea leonii* Chautems	EN
*Vanhouttea pendula* Chautems	EN
** Lentibulariaceae **	
*Genlisea lobata* Fromm	EN
*Utricularia tridentata* Sylvén	VU
** Melastomataceae **	
*Miconia setosociliata* Cogn.	VU
** Meliaceae **	
*Cedrela fissilis* Vell.	VU
*Cedrela odorata* L.	VU
** Monimiaceae **	
*Macropeplus schwackeanus* (Perkins) I.Santos & Peixoto	VU
** Orchidaceae **	
*Acianthera heringeri (Hoehne) F.Barros	CR
*Encyclia bragancae Ruschi	EN
*Epidendrum zappii* Pabst	EN
*Grandiphyllum divaricatum* (Lindl.) Docha Neto	VU
*Habenaria achalensis* Kraenzl.	VU
*Habenaria hydrophila* Barb.Rodr.	DD
*Phymatidium geiselii* Ruschi	EN
** Orobanchaceae **	
*Agalinis bandeirensis* Barringer	CR
*Nothochilus coccineus* Radlk.	EN
** Pentaphylacaceae **	
*Ternstroemia cuneifolia* Gardner	VU
** Plantaginaceae **	
*Achetaria caparaoense* (Brade) V.C.Souza	CR
** Poaceae **	
*Chusquea baculifera* Silveira	CR
*Chusquea heterophylla* Nees	EN
** Polygalaceae **	
*Polygala vollii* Brade	EN
** Primulaceae **	
*Myrsine villosissima* Mart.	EN
** Rubiaceae **	
*Psychotria paludosa* Müll.Arg.	EN
** Scrophulariaceae **	
*Buddleja longiflora* Brade	CR
*Buddleja speciosissima* Taub.	VU
** Smilacaceae **	
*Smilax lappacea* Willd.	EN
** Symplocaceae **	
*Symplocos itatiaiae* Wawra	EN
** Xyridaceae **	
*Xyris caparaoensis* Wand.	DD
*Xyris obtusiuscula* L.A.Nilsson	EN
** Zingiberaceae **	
*Renealmia brasiliensis K.Schum.	EN
**Ferns**	
** Anemiaceae **	
*Anemia blechnoides* Smith	VU
** Aspleniaceae **	
*Asplenium castaneum* Schltdl. et Cham.	EN
** Dicksoniaceae **	
*Dicksonia sellowiana* Hook.	EN
** Polypodiaceae **	
*Ceradenia capillaris* (Desv.) L.E.Bishop	VU
*Grammitis fluminensis Fée	EN
*Lellingeria tamandarei* (Rosenst.) A.R.Sm. & R.C.Moran	EN
*Pleopeltis monoides* (Weath.) Salino	EN
** Pteridaceae **	
*Doryopteris rediviva* Fée	VU
**Liverworts**	
** Arnelliaceae **	
*Gongylanthus liebmannianus* (Lindenb. & Gottsche) Steph.	EN
** Metzgeriaceae **	
*Metzgeria subaneura* Schiffn.	DD
** Pallaviciniaceae **	
*Jensenia difformis* (Nees) Grolle	EN
**Mosses**	
** Dicranaceae **	
*Atractylocarpus longisetus* (Hook.) E.B.Bartram	EN
** Ditrichaceae **	
*Chrysoblastella chilensis* (Mont.) Reimers	EN

**Table 2.** Rare species of plants occurring in “Parque Nacional do Caparaó,” Brazil and their respective families.

**Rare plant species**
** Apocynaceae **
*Oxypetalum leonii* Fontella
** Araceae **
*Anthurium mourae* Engl.
** Asteraceae **
*Baccharis dubia* Deble & A.S.Oliveira
*Chionolaena lychnophorioides* Sch.Bip.
*Leptostelma camposportoi* (Cabrera) A.M.Teles & Sobral
** Bromeliaceae **
*Dyckia bracteata* (Wittm.) Mez
*Neoregelia brownii* Leme
** Cactaceae **
*Schlumbergera kautskyi* (Horobin & McMillan) N.P.Taylor
** Eriocaulaceae **
*Paepalanthus caparoensis* Ruhland
** Gentianaceae **
*Senaea janeirensis* Brade
** Gesneriaceae **
*Vanhouttea leonii* Chautems
** Lentibulariaceae **
*Genlisea lobata* Fromm
** Monimiaceae **
*Macropeplus schwackeanus* (Perkins) I.Santos & Peixoto
** Myrtaceae **
*Siphoneugena delicata* Sobral & Proença
** Orchidaceae **
*Phymatidium geiselii* Ruschi
** Orobanchaceae **
*Agalinis bandeirensis* Barringer
*Esterhazya eitenorum* Barringer
*Nothochilus coccineus* Radlk.
** Piperaceae **
*Peperomia warmingii* C.DC.
** Polygalaceae **
*Polygala vollii* Brade
** Salicaceae **
*Abatia microphylla* Taub.
** Scrophulariaceae **
*Buddleja longiflora* Brade
*Buddleja speciosissima* Taub.
** Velloziaceae **
*Barbacenia irwiniana* L.B.Sm.
